# Elucidation of molecular kinetic schemes from macroscopic traces using system identification

**DOI:** 10.1371/journal.pcbi.1005376

**Published:** 2017-02-13

**Authors:** Miguel Fribourg, Diomedes E. Logothetis, Javier González-Maeso, Stuart C. Sealfon, Belén Galocha-Iragüen, Fernando Las-Heras Andrés, Vladimir Brezina

**Affiliations:** 1 Department of Neurology and Center for Translational Systems Biology, Icahn School Of Medicine at Mount Sinai, New York, New York, United States of America; 2 Department of Pharmaceutical Sciences, Northeastern University, Boston, Massachusetts, United States of America; 3 Department of Physiology and Biophysics, Virginia Commonwealth University School of Medicine, Richmond, Virginia, United States of America; 4 Department of Signals Systems and Radiocommunications, Universidad Politécnica de Madrid, Madrid, Spain; 5 Department of Electrical Engineering, Universidad de Oviedo, Gijón, Spain; 6 Department of Neuroscience, Icahn School of Medicine, New York, New York, United States of America; University of Leuven, UNITED STATES

## Abstract

Overall cellular responses to biologically-relevant stimuli are mediated by networks of simpler lower-level processes. Although information about some of these processes can now be obtained by visualizing and recording events at the molecular level, this is still possible only in especially favorable cases. Therefore the development of methods to extract the dynamics and relationships between the different lower-level (microscopic) processes from the overall (macroscopic) response remains a crucial challenge in the understanding of many aspects of physiology. Here we have devised a hybrid computational-analytical method to accomplish this task, the SYStems-based MOLecular kinetic scheme Extractor (SYSMOLE). SYSMOLE utilizes system-identification input-output analysis to obtain a transfer function between the stimulus and the overall cellular response in the Laplace-transformed domain. It then derives a Markov-chain state molecular kinetic scheme uniquely associated with the transfer function by means of a classification procedure and an analytical step that imposes general biological constraints. We first tested SYSMOLE with synthetic data and evaluated its performance in terms of its rate of convergence to the correct molecular kinetic scheme and its robustness to noise. We then examined its performance on real experimental traces by analyzing macroscopic calcium-current traces elicited by membrane depolarization. SYSMOLE derived the correct, previously known molecular kinetic scheme describing the activation and inactivation of the underlying calcium channels and correctly identified the accepted mechanism of action of nifedipine, a calcium-channel blocker clinically used in patients with cardiovascular disease. Finally, we applied SYSMOLE to study the pharmacology of a new class of glutamate antipsychotic drugs and their crosstalk mechanism through a heteromeric complex of G protein-coupled receptors. Our results indicate that our methodology can be successfully applied to accurately derive molecular kinetic schemes from experimental macroscopic traces, and we anticipate that it may be useful in the study of a wide variety of biological systems.

## Author summary

Unraveling the lower-level (microscopic) processes underlying the overall (macroscopic) cell response to a given stimulus is a challenging problem in cell physiology. This has been a classic problem in biophysics, where the ability to record the activity of single ion channels that generate a macroscopic ion current has allowed a measure of direct access to the underlying microscopic processes. These classic studies have demonstrated that very different groupings of the microscopic processes can yield extremely similar macroscopic responses. Biologists in fields other than biophysics are frequently confronted with the same macroscopic-to-microscopic problem, usually, however, without any direct access to the microscopic processes. Thus, the development of computational methods to deduce from the available macroscopic measurements the nature of the underlying microscopic processes can be expected to substantially advance the study of many areas of cell physiology. Toward that aim, here we have derived and tested a hybrid computational-analytical method to extract information about the microscopic processes that is hidden in macroscopic experimental traces. Our method is independent of the particular system under study, and thus can be applied to new as well as previously-recorded macroscopic traces obtained in a wide variety of biological systems.

## Introduction

In order to ensure their survival and correct physiological function, cells must respond appropriately to many different kinds of stimuli, such as the presence of various neurotransmitters or hormones in the extracellular fluid, the depolarization of the cell membrane, or stimuli such as mechanical force or light [1]. The cell’s response to any particular stimulus engages vast numbers of single molecules whose actions combine to perform various tasks within the cell.

**Fig 1:**
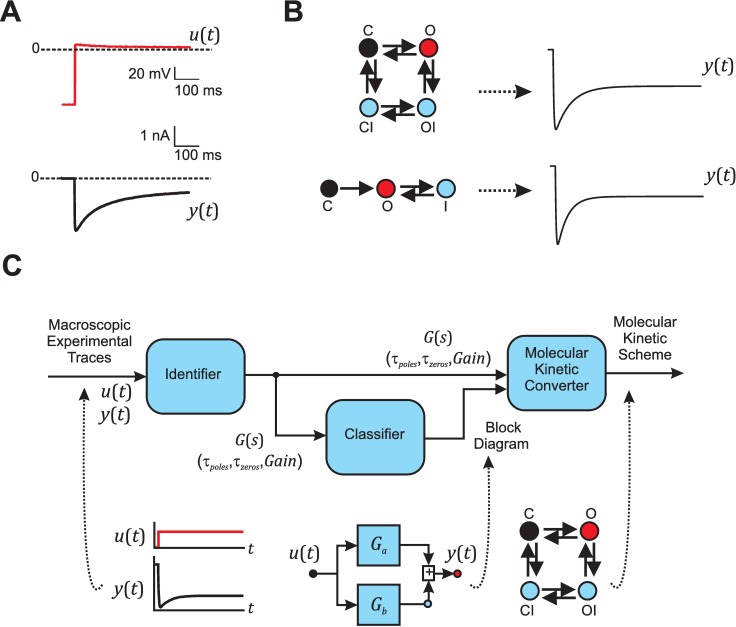
Problem description and modular implementation of SYSMOLE. (**A**) Example of classic voltage clamp experimental traces, which can be used as an input (red) and output (black) trace pair for the SYStems-based MOLecular kinetic scheme Extractor (SYSMOLE) method. In voltage-clamp (VC) experiments to study ion-channel currents, one would define an input-output system in which the scalar input signal *u*(*t*) is the depolarizing voltage step (top), and the scalar output signal *y*(*t*) is the actual current trace elicited by the depolarization (bottom). (**B**) Example of two molecular kinetic schemes describing ion channel dynamics in response to a voltage step that yield similar macroscopic traces. C indicates the closed-channel state, O the open-channel state, I the inactivated-channel state, CI the closed-inactivated-channel state, and OI the open-inactivated-channel state. (**C**) Modular organization of SYSMOLE and workflow of the method. Briefly, the Identifier Module uses the input *u*(*t*) and output *y*(*t*) traces to obtain the transfer function *G*(*s*), which can be characterized by the time constants of its poles (*τ*_*poles*_) and zeros (*τ*_*zeros*_), and a gain. The Classifier Module finds the configuration or block diagram associated with the transfer function *G*(*s*). The Molecular Kinetic Converter Module uses the block diagram together with the transfer function *G*(*s*) to derive the molecular kinetic scheme.

To provide a framework for the study of such cellular responses to stimuli, biologists try to understand the actions of the single molecules in terms of elementary (“microscopic”) molecular processes, such as the opening or closing of ion channels in the membrane, the phosphorylation or dephosphorylation of intracellular proteins, or the nuclear translocation of transcription factors [2, 3]. Formally, the dynamics of these processes are typically described by molecular kinetic schemes or, mathematically, Markov-chain state models. Each state in the model represents, for example, a molecular conformation or ligand binding-site occupancy; rate constants govern the transitions between the states (e.g., Fig 1). These types of schemes have been successfully applied to describe many kinds of cellular systems, including the activity of ion channels [4, 5], pharmacology of receptors [6–8], and flux through metabolic pathways [2].

Clearly, it is desirable to study these microscopic processes directly. This has been possible, to some extent, in a few areas, notably the biophysics of ion channels, an exceptionally favorable case where the exquisitely sensitive patch-clamp technique [9, 10] is able to record the activity of single ion channels. As a result, this technique provides some measure of direct information about microscopic processes such as the opening and closing of the channels and allows the construction of molecular kinetic models of these processes [11, 12].

In most areas of cell biology, however, such direct access to the microscopic processes is not yet possible. Although new single-molecule techniques provide ways of understanding the properties of key biological molecules in isolation, monitoring the dynamics of single molecules inside cells is technically complex and in many experimental systems not practical (see [13, 14] for reviews).

The information that *is* routinely available from cells exposed to stimuli is the overall (“macroscopic”) cellular response (e.g., Fig 1A). This response combines the signal from a large number of molecules and multiple microscopic processes [15, 16]. The macroscopic response trace thus contains information about the microscopic processes, and, since it is relatively easy to record in most experimental systems, an attractive approach is to attempt to extract information about the microscopic processes, and the way in which they are combined in the molecular kinetic scheme, from the macroscopic trace.

This, however, is a challenging problem. Although, in theory, the information about all of the relevant microscopic processes is present in the macroscopic trace, the trace combines the information about the individual processes in a way that is often complicated and difficult to interpret immediately in terms of the underlying molecular kinetic scheme [17]. For example, the work with ion channels has demonstrated that very similar macroscopic responses can be generated by quite different arrangements of the microscopic processes in the molecular kinetic scheme [18] (as can be seen also in Fig 1B).

One way to simplify the problem is to assume a pre-existing molecular kinetic model of the system, based on other kinds of experimental data or simply educated guesswork. Thus, in the ion-channel field, elegant computational methods have been introduced to analyze macroscopic traces of ion currents [19, 20], but these methods rely strongly on specific biophysical models of the ion channels and thus are not immediately generalizable to the study of other biological systems.

In this study, we have developed a general method, the SYStems-based MOLecular kinetic scheme Extractor (SYSMOLE), to obtain molecular kinetic schemes from macroscopic traces. SYSMOLE is a hybrid computational-analytical method that, because it does not assume any pre-existing model of the system, can be applied to experimental traces recorded, in principle, in any biological system.

In practical terms, to successfully derive the molecular kinetic scheme underlying a macroscopic trace, a method must perform three distinct tasks: a) it must accurately model the dynamics of the trace, b) it must ensure that this model is unique, and c) it must convert this mathematical model into a biologically interpretable model. SYSMOLE includes a module to tackle each of these tasks (Fig 1C). The Identifier Module accurately captures the dynamics of the macroscopic trace and summarizes this information in the form of a transfer function. The Classifier Module derives a block diagram, a description of how different processes are combined, that is uniquely matched to the transfer function. Finally, the Molecular Kinetic Converter (MKC) Module utilizes both the transfer function and the block diagram to derive a molecular kinetic scheme that can be interpreted biologically in terms of conformational changes of proteins or chemical reactions.

We first describe the implementation of each of the three modules that comprise SYSMOLE. For didactic simplicity, we focus in our presentation on a second-order system, in which two first-order processes are combined in different arrangements; however, our approach is scalable to higher-order systems (S1 Text section 3). Next, we provide a rigorous evaluation of the performance of SYSMOLE in the presence of noise using synthetic traces. We then report on the performance of SYSMOLE on real experimental traces. We show that SYSMOLE was able to derive the correct molecular kinetic scheme of the inactivation of L-type calcium channels as well as the effects of the calcium-channel blocker nifedipine from macroscopic calcium-current traces. Finally, we describe the use of SYSMOLE to decipher the crosstalk mechanism, through the heteromeric receptor complex formed by the metabotropic glutamate receptor 2 (mGluR2) and the serotonin receptor 2A (5-HT2AR), of a new class of glutamate antipsychotics.

## Results

### Identifier module

#### Description

SYSMOLE aims to understand the system’s response to stimuli by breaking it down in terms of simple processes (i.e. activation, or inactivation). To that aim SYSMOLE relies on System Theory, which has developed robust process-oriented approaches using transformed domains, such as Fourier, Laplace or z, as an alternative functional description that simplifies the process of analyzing the behavior of the system. Laplace transformation from the time domain (*t*) to the frequency domain (*s*), for example, transforms differential equations into algebraic equations and convolution into multiplication, which simplifies the analysis. Furthermore, it allows for a description of the system’s response in the frequency domain as the product *Y*(*s*) = *G*(*s*). *U*(*s*), where *Y*(*s*) and *U*(*s*) refer to the output and the input signals in the frequency domain respectively, and *G*(*s*) to the transfer function that characterizes the system. The transfer function provides in principle the output for any input within certain constraints (see below). Despite this input-output characterization not being suited for systems with multiple alternative outputs to one input such as bistable systems, it provides an extremely powerful characterization of the response to stimuli of a wide range of systems.

The Identifier Module embodies the system-identification component of our approach. It uses the relationship between a given input trace *u*(*t*) and the resulting output trace *y*(*t*) to characterize the dynamics of the system and to summarize them in the form of an overall transfer function *G*(*s*).

#### Implementation

The Identifier Module considers systems to be causal, linear, and time-invariant (LTI). Causality implies that the output at a certain time depends only on the input up to that time. Linearity enforces the output response of the system to a linear combination of inputs to be equal to the linear combination of the output responses to the individual inputs. Time-invariance means that the output response to a given input does not depend on absolute time. Experimentally, these assumptions can be met by applying some simple constraints. To ensure linearity, one can expose the system to a range of inputs known not to saturate the system. To ensure time-invariance, one can wait for the system to be at steady state before exposing it to the input and measure its response only once if long-term desensitization or other long-term memory processes are known to play a significant role in the system. Given this LTI characterization, SYSMOLE is best adapted to study systems whose behavior can be described or approximated by ordinary differential equations (ODEs).

How does SYSMOLE derive the transfer function in the frequency domain, *G*(*s*), from the input *u*(*t*) and output *y*(*t*) traces in the time domain? It is well known (see [21] for a review) that a LTI system can be described by its impulse response *g*(*t*) as follows:

$$y(t)=\int_{\tau =0}^{\infty }g(\tau )u(t-\tau )d\tau$$ (1)

Thus, knowing *g*(*t*) from *t* = 0 to ∞, one can compute the output *y*(*t*) for any input *u*(*t*). The impulse response *g*(*t*) thus provides a complete characterization of the system. The transfer function *G*(*s*) is the representation of *g*(*t*) in the Laplace-transformed domain. Both time *t* and the transformed variable *s* are continuous; in real experiments, however, both *u*(*t*) and *y*(*t*) are sampled discretely in time. The Identifier Module assumes that *u*(*t*) and *y*(*t*) are observed at the sampling instants *t*_*k*_ = *kT*_*s*_ with *k* = 1,2,…, where *T*_*s*_ is the sampling interval. In those terms one can rewrite Eq (1) as

$$y(t)=\int_{\tau =0}^{\infty }g(\tau )u(t_{k}-\tau )d\tau$$ (2)

Assuming that *u*(*t*) does not change much during a sample interval one can express *y*(*t*) as

$$y(t)=\sum_{k=1}^{\infty }g(k)u(t-k)$$ (3)

where we ease the notation and assume that *T*_*s*_ is one time unit and use *t* to enumerate the sampling intervals.

According to relationship (3) the output can be exactly calculated once the input is known. In most cases this is unrealistic, since there are always signals beyond our control that also affect the system. In the linear framework used by the Identifier Module those effects are lumped into an additive term at the output *v*(*t*) called disturbance.

$$y(t)=\sum_{k=1}^{\infty }g(k)u(t-k)+v(t)$$ (4)

Inputs could also be corrupted but their effects are included on the output in *v*(*t*) without loss of generality. Disturbances can come from measurement noise, and other uncontrollable inputs [22]. The most characteristic feature of the disturbance is that its value is not known beforehand, and therefore one requires a probabilistic framework to characterize it. The Identifier Module mathematically expresses *v*(*t*) as the output of a system characterized by the discrete-time impulse response *h*(*k*)

$$v(t)=\sum_{k=0}^{\infty }h(k)e(t-k)$$ (5)

where the input *e*(*t*) is white gaussian noise with zero mean and variance *λ*. Although *e*(*t*) being Gaussian is a somewhat restraining assumption, it still allows us to characterize a wide range of disturbances *v*(*t*) by using different *h*(*k*) sequences. This model of disturbance is extremely versatile and should be appropriate to characterize the noise present in a wide range of experimental traces [23]. We have successfully applied it to traces with added Gaussian noise (see next sections), and Brownian noise (S1 Text section 4.1)

In summary, as part of the identification process, the Identifier Module will find the discrete-time response *g*(*k*) and the discrete-time transfer function *G*(*z*) that relates the discrete input and the discrete output. The continuous version of the transfer function *G*(*s*) is obtained by converting *G*(*z*) to the continuous domain *G*(*s*). Furthermore, as part of the identification process the Identifier Module will obtain *H*(*s*), the Laplace Transform of *h*(*k*), which generates the disturbance by filtering white Gaussian noise.

The dynamics of a system can be expressed in many different ways. Probably the simplest input-output relationship between two discrete-time signals is obtained by describing it as a linear difference equation. The Identifier Module describes this relationship as an ARX model (6), a family of difference equations characterized by a set of parameters $\theta =[\alpha_{1},\alpha_{2},\ldots ,\alpha_{n_{\alpha }},\beta_{1},\beta_{2},\ldots ,\beta_{n_{\beta }}]$.

$$y(t)+\alpha_{1}y(t-1)+\cdots +\alpha_{n_{a}}y(t-n_{\alpha })=\beta_{1}u(t-1)+\cdots +\beta_{n_{\beta }}u(t-n_{\beta })+e(t)$$ (6)

Here AR refers to the autoregressive part of *y*(*t*) and X to the contribution of the input *u*(*t*) at earlier times, also called the exogenous variable. We chose the ARX models for the implementation of the identifier for two main reasons: (a) the best parameter estimation given the disturbance can be easily obtained computationally [24], and (b) the parameters of the model can be easily converted to the poles and zeros of the transfer function *G*(*s*). The Identifier Module determines optimal values of *n*_*α*_, *n*_*β*_ as well as the coefficients $\alpha_{1},\ldots ,\alpha_{n_{a}}$ and $\beta_{1},\ldots ,\beta_{n_{\beta }}$ that minimize the prediction error in the model by minimizing a function denoted loss function (see prediction-error identification methods in [25]). Our identifications provided loss functions of the order 10^−7^ or lower indicating that ARX models and Eq (6) provide good fits to the synthetic and experimental traces we studied.

It can be also demonstrated that *n*_*α*_ − 1 represents the number of poles, and *n*_*β*_ the number of zeros of the discrete transfer function *G*(*z*). Furthermore, by adequately sampling the traces, we can convert the transfer function obtained in the discrete frequency domain, *G*(*z*), to its equivalent transfer function in the continuous frequency domain, *G*(*s*) [26]. Each transfer function *G*(*s*) is characterized by the roots of the denominator (poles), the roots of the numerator (zeros), and the gain. The minimum sampling frequency is given by the Nyquist-Shannon sampling theorem applied to the power density spectrum of the signal *y*(*t*), and empirically estimated in a conservative fashion by choosing a sampling period *T*_*s*_ one or two orders of magnitude below the smaller time constant.

### Classifier module

#### Description

**Fig 2:**
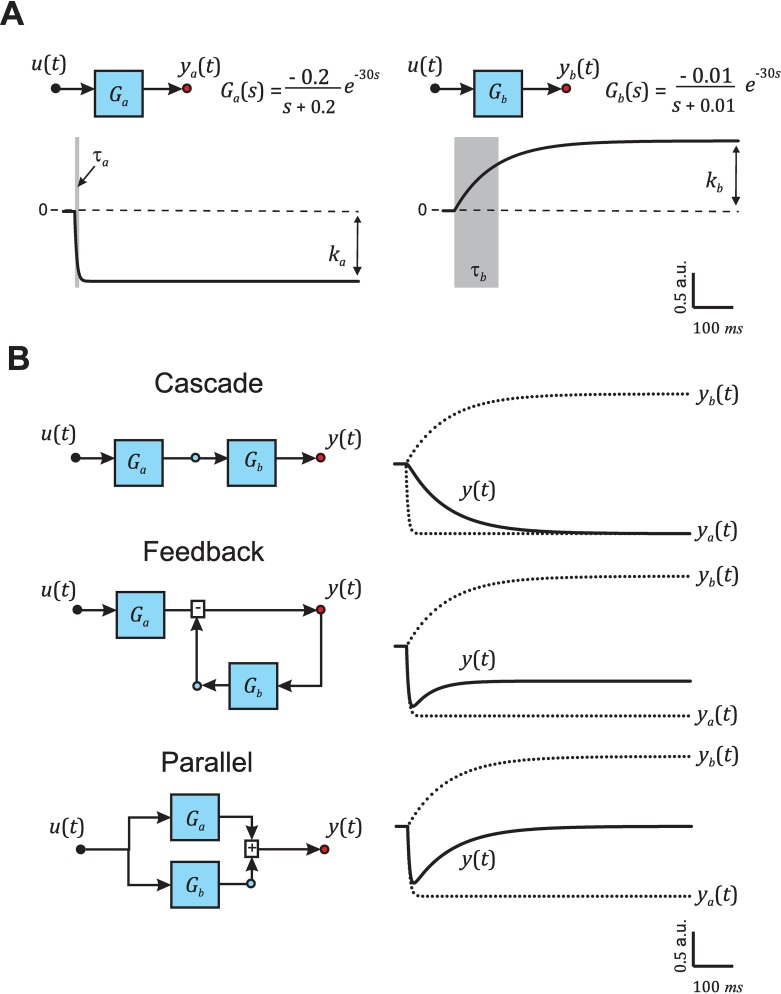
Second-order canonical configurations. (**A**) Example of two first-order processes, *a* and *b*, described by first-order transfer functions *G*_*a*_(*s*) and *G*_*b*_(*s*) with parameters *τ*_*a*_ = 5 ms and *k*_*a*_ = -1, and *τ*_*b*_ = 100 ms and *k*_*b*_ = 1, and their responses in time (outputs *y*_*a*_(*t*) and *y*_*b*_(*t*)) to an ideal step input at *t* = 30 ms (*u*(*t*)). (**B**) Block diagram (left) and corresponding output *y*(*t*) (right) in response to a step input *u*(*t*) of the first-order processes *G*_*a*_(*s*) and *G*_*b*_(*s*) combined following the three canonical configurations: cascade, feedback and parallel.

In the Laplace frequency domain used by SYSMOLE, each process *i* is described by a first-order system (associated with a first-order ODE), with a transfer function *G*_*i*_(*s*) characterized by a gain parameter *k*_*i*_ and a time constant *τ*_*i*_ as follows:

$$G_{i}(s)=\frac{b_{i}}{s+\omega_{i}}$$ (7)

where $\omega_{i}=\frac{1}{\tau_{i}}$ and $b_{i}=\frac{k_{i}}{\tau_{i}}$. Given two processes *a* and *b* described by first-order systems, and thus by first-order transfer functions *G*_*a*_(*s*) and *G*_*b*_(*s*) (e.g., those in Fig 2A) one can find only three truly different ways to combine them: cascade, feedback, and parallel (Fig 2B). We will refer to these configurations as canonical, since any combination of two first-order processes can be converted to one of these three configurations with a simple change in parameters [27, 28]. Each canonical configuration has a corresponding block diagram as defined in Systems Theory [23] (Fig 2B, left) and yields a response with different dynamics (Fig 2B, right). Combining two first-order processes will result in a second-order transfer function characterized by two poles and one or no zeros. The dependence of the poles, zeros, and gain of *G*(*s*) on the parameters *τ*_*a*_, *τ*_*b*_, *k*_*a*_, and *k*_*b*_ of the two first-order processes differs greatly between the three canonical configurations and provides each configuration with a characteristic signature (S1 Fig).

Given two first-order systems *G*_*a*_(*s*) and *G*_*b*_(*s*) described by first-order transfer functions $G_{a}(s)=\frac{b_{a}}{s+\omega_{a}}$ and $G_{b}(s)=\frac{b_{b}}{s+\omega_{b}}$, one can analytically derive the transfer functions resulting from combining these two processes following each canonical configuration and obtain:

$${G(s)}_{cascade}=\frac{b_{a}b_{b}}{\left(s+\omega_{a})(s+\omega_{b}\right)}$$ (8)

$${G(s)}_{feedback}=\frac{b_{a}(s+\omega_{b})}{\left(s+\omega_{a})(s+\omega_{b}+b_{b}\right)}$$ (9)

$${G(s)}_{parallel}=\frac{{(b_{a}+b_{b})s+b}_{a}\omega_{b}+b_{b}\omega_{a}}{\left(s+\omega_{a})(s+\omega_{b}\right)}$$ (10)

As discussed above, each of these transfer functions has distinct features in terms of poles, zeros, and gain (*τ*_*pole*1_, *τ*_*pole*2_, *τ*_*zero*_, and *Gain*). The Classifier Module capitalizes on these differences and determines which canonical configuration corresponds to the transfer function obtained by the Identifier Module, *G*(*s*), by comparing the transfer function features of *G*(*s*) to those of the canonical *G*(*s*)_*cascade*_, *G*(*s*)_*feedback*_, and *G*(*s*)_*parallel*_.

#### Implementation

**Fig 3:**
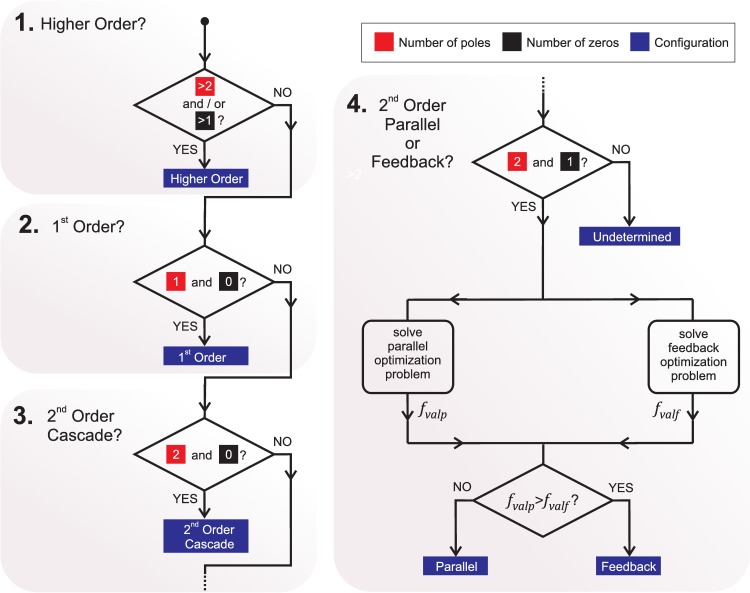
Classifier Module implementation. Classifier Module flow chart implementation of the comparison between *G*(*s*), the transfer function obtained by the Identifier Module, and the transfer functions associated with the canonical configurations. This module tests sequentially whether *G*(*s*) incorporates the features of a transfer function describing a higher-order system, a first-order system, or a second-order system in each of the canonical configurations: cascade, feedback or parallel. The cost function values for the parallel and feedback optimization problems, *f*_*valp*_ and *f*_*valf*_ respectively, are used to discern between these two canonical configurations as explained in the *Results* section.

The Classifier Module is implemented as a series of comparisons between *G*(*s*) and the canonical transfer functions (8), (9) and (10) (Fig 3). It tests sequentially whether the transfer function features for a particular configuration are found in *G*(*s*). Transfer functions arising from systems with more than two processes are characterized by more than two poles. Similarly, first-order transfer functions are characterized by having only one pole and no zeros and can be easily distinguished. From the second-order transfer functions, characterized by two poles, those arising from a cascade configuration have no zeros and are easily classified as well. However, discerning between parallel and feedback configurations constitutes a more challenging task.

It can be mathematically demonstrated that for every transfer function *G*(*s*) with two poles and one zero arising from two fist-order processes, one can find a set of first-order systems *G*_*af*_(*s*) and *G*_*bf*_(*s*) which, when combined in feedback, will result in *G*(*s*). It can also be demonstrated that one can find an analogous set of first-order systems *G*_*ap*_(*s*) and *G*_*bp*_(*s*) which, when combined in parallel, will result *G*(*s*) (S1 Text). To distinguish between the two configurations one needs to constrain the problem by imposing additional conditions on the possible values of *τ*_*a*_, *τ*_*b*_, *k*_*a*_, and *k*_*b*_. Typically, we are able to provide a range for the speed and strength of each process under study and therefore adding this type of constraint does not constitute a complex task. As an example, from the trace depicted on the introductory figure (Fig 1A, bottom), one can easily constrain the activation process (fast downward inflection) to have a time constant ranging from 1 to 50 *ms*, and the inactivation process (slow upward inflection) to have a time constant greater than 50 *ms*. Similarly, one can restrict the exploration of the parameter space to activation and inactivation gains between -0.2 to 0.2 nA/mV.

The Classifier Module then decides between the feedback and parallel configurations by solving two constrained optimization problems (Fig 3). These two problems are defined by comparing the second-order transfer function *G*(*s*) characterized by a 4-parameter set, either (*τ*_*pole*1_, *τ*_*pole*2_, *τ*_*zero*_, and *Gain*) or (*B*0, *B*1, *A*1, and *A*0),

$$G(s)=\frac{B_{1}s+B_{0}}{s^{2}+A_{1}s+A_{0}}$$ (11)

to *G*(*s*)_*feedback*_ and *G*(*s*)_*parallel*_. We define the feedback optimization problem by comparing (11) to (9). Solving the problem thus amounts to determining whether there is a solution for the following system of equations within the range of values allowed for the parameters *ω*_*a*_, *ω*_*b*_, *b*_*a*_, and *b*_*b*_

$$\left\{ \begin{array}{c} B_{1}=b_{a}\\ B_{0}=b_{a}\omega_{b}\\ \begin{array}{c} A_{1}=\omega_{a}+\omega_{b}+b_{b}\\ A_{0}=\omega_{a}(\omega_{b}{+b}_{b}) \end{array} \end{array}\right.$$ (12)

Computationally we solve this problem as an optimization problem in which we minimize the quadratic cost function derived from the system of equations in (12)

$$f_{valf}(b_{a},b_{b},\omega_{a}.\omega_{b})=\alpha_{1}{\left|B_{1}-b_{a}\right|}^{2}+\alpha_{2}{\left|B_{0}-b_{a}\omega_{b}\right|}^{2}+\cdots \cdots +\alpha_{3}{\left|A_{1}-(\omega_{a}+\omega_{b}+b_{b})\right|}^{2}+\alpha_{4}{\left|A_{0}-\omega_{a}(\omega_{b}+b_{b})\right|}^{2}$$ (13)

subjected to the constraints, *ω*_*amin*_ ≤ *ω*_*a*_ ≤ *ω*_*amax*_, *ω*_*bmin*_ ≤ *ω*_*b*_ ≤ *ω*_*bmax*_, *b*_*amin*_ ≤ *b*_*a*_ ≤ *b*_*amax*_, and *b*_*bmin*_ ≤ *b*_*b*_ ≤ *b*_*bmax*_.

We define analogously the parallel optimization problem. Given the transfer function for the parallel system

$$G(s)=\frac{(b_{a}+b_{b})s+b_{a}\omega_{b}+b_{b}\omega_{a}}{\left(s+\omega_{a})(s+\omega_{b}\right)}$$ (14)

and the equivalent system of equations

$$\left\{ \begin{array}{c} B_{1}=b_{a}+b_{b}\\ B_{0}=b_{a}\omega_{b}+b_{b}\omega_{a}\\ \begin{array}{c} A_{1}=\omega_{a}+\omega_{b}\\ A_{0}=\omega_{a}\omega_{b} \end{array} \end{array}\right.$$ (15)

we can computationally find the solution as an optimization problem in which we minimize the quadratic cost function

$$f_{valp}(b_{a},b_{b},\omega_{a}.\omega_{b})=\alpha_{1}{\left|B_{1}-(b_{a}+b_{b})\right|}^{2}+\alpha_{2}{\left|B_{0}-(b_{a}\omega_{b}+b_{b}\omega_{a})\right|}^{2}+\cdots \cdots +\alpha_{3}{\left|A_{1}-(\omega_{a}+\omega_{b})\right|}^{2}+\alpha_{4}{\left|A_{0}-\omega_{a}\omega_{b}\right|}^{2}$$ (16)

subjected to the constraints, *ω*_*amin*_ ≤ *ω*_*a*_ ≤ *ω*_*amax*_, *ω*_*bmin*_ ≤ *ω*_*b*_ ≤ *ω*_*bmax*_, *b*_*amin*_ ≤ *b*_*a*_ ≤ *b*_*amax*_, and *b*_*bmin*_ ≤ *b*_*b*_ ≤ *b*_*bmax*_.

The cost function values *f*_*valf*_ and *f*_*valp*_ provide a quantitative measure of the match between *G*(*s*) and *G*(*s*)_*feedback*_, and *G*(*s*)_*parallel*_ respectively, provided that the constraints are sufficient to distinguish both processes (S1 Text). A value of *f*_*valf*_ greater than *f*_*valp*_ indicates that *G*(*s*) arose from two processes combined in parallel. Conversely, a value of *f*_*valp*_ greater than *f*_*valf*_ indicates a transfer function *G*(*s*) associated with two processes combined following the feedback configuration (Fig 3). We studied the convergence properties of these two optimization problems for a large number of combinations and a wide value range for parameters *τ*_*a*_, *τ*_*b*_, *k*_*a*_, and *k*_*b*_. Our results confirmed the robustness of this implementation to discern between parallel and feedback configurations (S2 Fig). Similar principles can be applied to third-order systems by including more optimization problems in the implementation (see S1 Text section 3 for a description of a Classifier Module for transfer functions with three poles and two zeros).

### Molecular kinetic converter

#### Description

In general, obtaining a molecular kinetic scheme associated with a given transfer function *G*(*s*) represents an underdetermined problem with multiple possible solutions [18]. Here we show we can use biological principles and our prior information about the biological system under study to constrain the problem and obtain a unique solution. This task, which is analytical as opposed to computational, is implemented by the Molecular Kinetic Converter Module (MKC). Implementing the biological constraints in mathematical/analytical terms is not straightforward. The MKC is implemented as a set of rules or steps that impose different biological/physical principles on the transfer function *G*(*s*) obtained by the Identifier Module, and are based on the block diagram (configuration) derived by the Classifier Module for that particular transfer function. It should be emphasized that the MKC strictly derives a Markov-chain state model and that the interpretation of what each state represents in molecular terms will depend on the experimental conditions in which traces are obtained.

#### Implementation

Our methodology converts the *G*(*s*) and block diagram (configuration) into a Markov-chain molecular kinetic scheme by implementing the following four principles:

1. *Number of states and possible transitions*. The number of processes that can be extracted from the input and output traces is established by the order *n* of the transfer function *G*(*s*). The relationship between these processes in the biological system, which is determined by the configuration, defines the possible transitions in the system.
2. *Mass conservation*. Mass should be conserved and thus the total amount of molecules, which is the sum of the occupancies of all the states in the system, has to remain constant. Importantly, although ideally all possible states should be described, additional biologically possible states not detected by our experimental assay might be lumped into one state. Furthermore, if one of the processes is removing a given species from the system, through degradation for example, a ‘degraded’ state will need to be included to satisfy this condition.
3. *Microscopic reversibility*. Corresponding to every individual set of transitions that takes a molecule through several states back to its original state (forward drive), there is a reverse set of transitions (reverse drive) such that in a state of equilibrium the average rate of the forward drive is equal to the average rate of its reverse drive. In other words, although the macroscopic law of mass action reactions can be used to describe processes that perpetually circle through states, at the molecular level the microscopic reversibility principle states that molecules do not possess infinite energy to remain in constant motion. In our case, a constant circular flow of transitions involving three or more states would contradict this principle and thus would result in a molecular kinetic scheme not biologically possible [6, 29, 30].
4. *Observable state*. Our experimental assay will typically only allow us to measure one of the states of the biological system, such as the open state of a channel or the activated form of a protein. This observable state should be identified since its occupancy as a function of time is related directly to the measured value of the output signal *y*(*t*).

The molecular kinetic scheme associated with the transfer function *G*(*s*) which satisfies the previous four principles can be derived as follows:

1. Identify the nodes in the block diagram. The number of states (*N*_*s*_) is equal to the number of nodes and equal to *n* + 1. In the context of this analytical tool, nodes are the points in the block diagram located at the entrance and at the exit of each block (operation blocks like addition and subtraction only add one node). It should be noted that these rules provide the molecular kinetic scheme with the smallest number of states to represent the number of detected first-order processes *n*. More states might exist at the microscopic level that would be collapsed into one of the states. The fractional occupancy in each state *i* is described by a time-dependent state variable *z*_*i*_(*t*) where *i* = 1 … *n* + 1.
2. Include *r* transitions between nodes for each connection described in the block diagram with rates *σ*_*k*_ with *k* = 1 … *r*. The value of the signal at a node in a block diagram can be negative, however negative fractional occupancies of a state are not interpretable. In order to implement negative values and subtractions in the molecular kinetic scheme, invert the flow of the signal and the transition (Fig 4).

**Fig 4:**
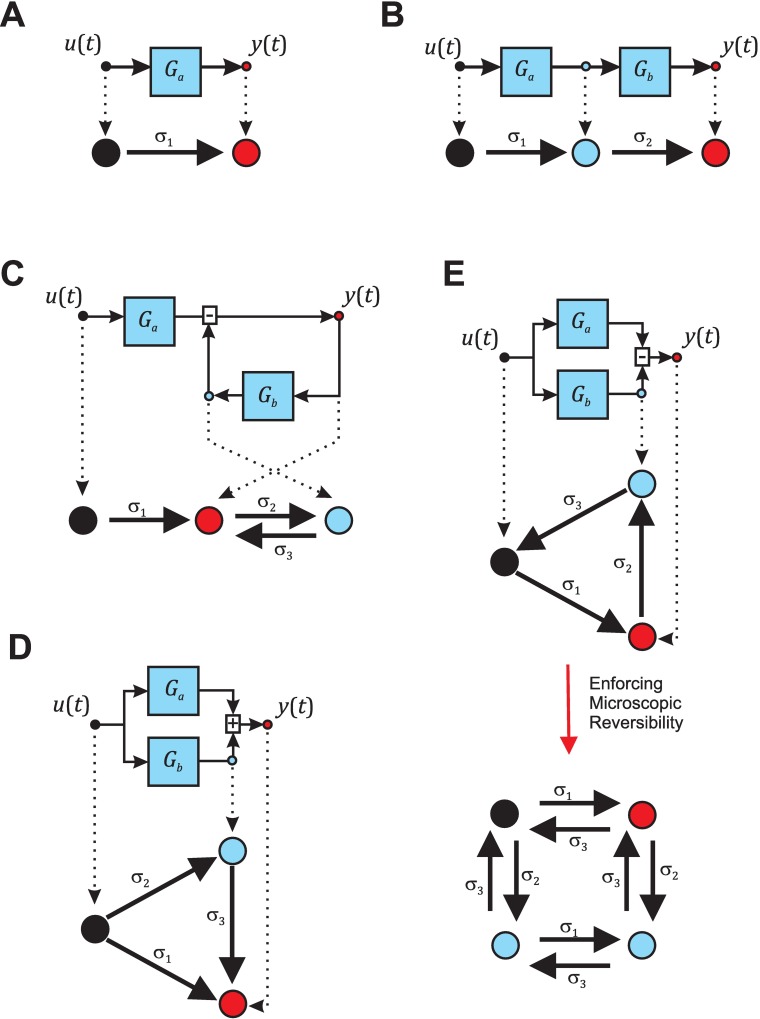
Molecular Kinetic Converter Module implementation. Molecular kinetic schemes derived from block diagrams for first-order systems (**A**), second-order systems in cascade (**B**), second-order systems in feedback (**C**), second-order in parallel addition (**D**), and second-order systems in parallel subtraction (**E**) configurations. Red circles indicate the observable state for each configuration. Black and blue circles depict non-observable states. The supporting material (S1 Text) provides, based on the steps described in the main text, a complete derivation of the molecular kinetic scheme corresponding to each configuration. It also includes, for each configuration, the equations that describe the transition rates between states (*σ*_*k*_) and the observable proportionality constant (*γ*) as a function of the pole and zero time constants (*τ*_*pole1*_, *τ*_*pole*2_, and *τ*_*zero*_), and the gain of the system.
3. Check whether the principle of microscopic reversibility is satisfied by the molecular kinetic scheme. If any perpetual circular flows involving three or more states are present, offset it by adding an equivalent flow in the opposite direction using only the kinetic parameters of the system *σ*_*k*_, to avoid increasing the number of degrees of freedom *r*.
4. Generate the following system of equations in the time domain
  - *N*_*s*_ − 1 Ordinary Differential Equations (ODEs) describing the transitions between the states of the form
    $$\frac{{dz}_{i}(t)}{dt}=\sum_{x=1}^{l}\sigma_{x}z_{x}(t)-\sum_{y=1}^{m}\sigma_{y}z_{y}(t)\;i=1\;\ldots \;N_{s}-1$$ (17)
    where *l* is the number of transitions coming into the state *i*, and *m* is the number of transitions leaving the state *i*. These equations follow the hypothesis of a Markov chain (i.e. the rates *σ* are independent of time and the state variables *z*_*i*_(*t*)) and can be assimilated to Kirchhoff’s First Law for circuit analysis.
  - 1 equation relating the observable state variable *z*_*obs*_(*t*) to the output *y*(*t*) through a proportionality constant *γ* (Observable Equation).
    $$y(t)=\gamma z_{obs}(t)$$ (18)
    Although for the experimental traces analyzed in the present work a simple proportionality constant provided was a sufficient model, it is not necessary for the output to strictly be proportional to the observation. If the relationship between the observed state and the measurement is known and can be characterized by another transfer function in the frequency domain *γ*(*s*) this can be included in the relationship (18).
  - 1 equation that establishes mass conservation. It is hard to implement the input signal in Markov chain models. Through the mass-conservation equation we can include the input *u*(*t*) as the unique force driving the change in number of molecules in each state as a function of time *z*_*i*_(*t*) of the system that we assume to be initially at steady state. By enforcing
    $$u(t)=\sum_{i=1}^{n+1}z_{i}(t)$$ (19)
    we guarantee that the total number of molecules in the system is equal to the total amount of molecules before the input, together with the molecules provided by the input. This also ensures that molecules are neither created nor destroyed. For an input step, as the ones *u*(*t*) is a constant that we can normalize to 1, and *z*_*i*_(*t*) can be interpreted as fractional occupancies in the classic ion channel sense. For simplicity, and given that this study focuses on experimental systems in which the input is a step, we will refer to *z*_*i*_(*t*) as fractional occupancies hereinafter. This can however be extended to inputs with any type of dynamics, which is one of the strengths of the method. It should be emphasized that the principles of mass conservation and microscopy reversibility are only imposed on the state fractional occupancies and transition rates which are affected by the particular stimulus or input under study. Other states whose fractional occupancies remain constant or at steady-state could exist, but will not be included in the description.
5. Laplace transform the system of equations
  $$\left\{ \begin{array}{c} sZ_{i}(s)-z_{i}(0)=\sum_{x=1}^{l}\sigma_{x}Z_{x}(s)-\sum_{y=1}^{m}\sigma_{y}Z_{y}(s)\;i=1\;\ldots \;n\\ Y(s)=\gamma Z_{obs}(s)\\ U(s)=\sum_{i=1}^{n+1}Z_{i}(s) \end{array}\right.$$ (20)
  where *z*_*i*_(0) is the value of the state variable at the initial time. Typically, one can choose the states where *z*_*i*_(0) = 0 to be in the differential equations to simplify the resolution of the system. The “input” state, in which the system has initially a fractional occupancy of 1, is then included in the mass conservation equation. It should be noted however that the approach can be applied for any initial distribution of fractional occupancies amongst states.
6. To obtain the transfer function of the kinetic model *G*(*s*)_*kin*_ we isolate
  $$\frac{Y(s)}{U(s)}={G(s)}_{kin}=\frac{C\;\prod_{j=1}^{n-1}\left(s+D_{j}\right)}{\prod_{j=1}^{n}\left(s+A_{j}\right)}$$ (21)
  where *C*, *D*_*j*_ and *A*_*j*_ are constants.
7. Using block algebra we obtain the transfer function in terms of the first-order systems
  $$G(s)=\frac{c\;\prod_{j=1}^{n-1}\left(s+d_{j}\right)}{\prod_{j=1}^{n}\left(s+a_{j}\right)}$$ (22)
  where *c*, *d*_*j*_ and *a*_*j*_ are constants (see Eqs 8, 9 and 10 for examples).
8. Solve the following system of equations
  $$\left\{ \begin{array}{l} D_{j}=d_{j}\;j=1\;\ldots \;n-1\\ A_{j}=a_{j}\;j=1\;\ldots \;n\\ \;{{G(s)}_{kin}\rfloor }_{s\to 0}={G(s)\rfloor }_{s\to 0} \end{array}\right.$$ (23)
  where the third equation in the system equals the steady state gain of *G*(*s*) and *G*(*s*)_*kin*_, for the parameters of the molecular kinetic scheme
  $$\sigma_{k}=f(k_{i},\omega_{i})$$ (24)
  $$\gamma =f(k_{i},\omega_{i})$$ (25)

It is straightforward to test that these rules yield the correct molecular kinetic scheme as any mistake in the steps will result in a system of equations without solutions at step 8 when comparing the coefficients on *G*(*s*)_*kin*_ and the *G*(*s*) derived from the block algebra (S1 Text section 2.5). As an illustration of the application of these steps, we present below the derivation the molecular kinetic scheme associated with a first-order system (Fig 4A):

1. Number of states = *n* + 1 = 2
2. We identify the nodes and include the transitions in the diagram.
3. We check that the principle of microscopic reversibility is satisfied.
4. We build the system of equations
  $$\left\{ \begin{array}{l} \frac{dz_{2}(t)}{dt}=\sigma_{1}z_{1}(t)\;\mathrm{ODE}\;\mathrm{Transition}\;1\\ y(t)=\gamma z_{2}(t)\;\mathrm{Observable}\;\mathrm{Equation}\\ \;u(t)=z_{1}(t)+z_{2}(t)\;\mathrm{Mass}\;\mathrm{Equation} \end{array}\right.$$ (26)
5. We Laplace transform the system
  $$\left\{ \begin{array}{l} sZ_{2}(s)=\sigma_{1}Z_{1}(s)\;T1\\ \;Y(s)=\gamma Z_{2}(s)\;O\\ U(s)=Z_{1}(s)+Z_{2}(s)\;M \end{array}\right.$$ (27)
6. We obtain *G*(*s*)_*kin*_ by isolating $\frac{Y(s)}{U(s)}$ from equations T1, O and M.
  $${G(s)}_{kin}=\frac{Y(s)}{U(s)}=\frac{\sigma_{1}\gamma }{s+\sigma_{1}}$$ (28)
7. From the block diagram we obtain *G*(*s*)
  $$G(s)=\frac{b_{a}}{s+\omega_{a}}$$ (29)
8. Through comparison of the terms in *G*(*s*)_*kin*_ and *G*(*s*) we obtain the parameters of the molecular kinetic scheme
  $$\sigma_{1}=\omega_{a}$$ (30)
  $${{G(s)}_{kin}\rfloor }_{s\to 0}=\gamma ={G(s)\rfloor }_{s\to 0}=\frac{b_{a}}{\omega_{a}}\Rightarrow \gamma =\frac{b_{a}}{\omega_{a}}$$ (31)

A complete description of the application of these steps to derive the molecular kinetic schemes corresponding to the three possible canonical configurations for second-order systems (Fig 4B, 4C, 4D and 4E) is provided in the supporting material (S1 Text). Indications on how these steps could be easily scaled to higher-order systems by taking a module-based approach are also provided. The values of the kinetic parameters *σ*_*i*_ and *γ* of the four possible second-order molecular kinetic schemes are detailed below. It is important to note that, based on step 2, the parallel configuration will yield two different molecular kinetic schemes depending on whether the first-order systems *G*_*a*_ and *G*_*b*_ are added or subtracted.

1. Second-order system in cascade configuration (Fig 4B)
  $$\sigma_{1}=\omega_{a}$$ (32)
  $$\sigma_{2}=\omega_{b}$$ (33)
  $$\gamma =k_{a}k_{b}$$ (34)
2. Second-order system in feedback configuration (Fig 4C)
  $$\sigma_{1}=\omega_{a}$$ (35)
  $$\sigma_{2}=b_{b}$$ (36)
  $$\sigma_{3}=\omega_{b}$$ (37)
  $$\gamma =k_{a}$$ (38)
3. Second-order system in parallel addition configuration (Fig 4D)
  $$\sigma_{1}=\frac{b_{a}+b_{b}}{k_{a}+k_{b}}$$ (39)
  $$\sigma_{2}=\omega_{b}-\sigma_{1}$$ (40)
  $$\sigma_{3}=\omega_{a}$$ (41)
  $$\gamma =k_{a}+k_{b}$$ (42)
4. Second-order system in parallel subtraction configuration (Fig 4E)
  $$\sigma_{1}=\omega_{a}$$ (43)
  $$\sigma_{2}=\omega_{b}-\sigma_{3}$$ (44)
  $$\sigma_{3}=\frac{b_{a}\omega_{b}+b_{b}\omega_{a}}{b_{a}+b_{b}}$$ (45)
  $$\gamma =\frac{(k_{a}+k_{b})\omega_{a}\omega_{b}}{\sigma_{3}}$$ (46)

### SYSMOLE distinguishes different molecular kinetic schemes from similar macroscopic traces

We decided to test first the ability of SYSMOLE to tackle a general class of problems in biology: distinguishing whether two similar macroscopic traces arise from different molecular kinetic schemes. Elucidating the molecular mechanisms of ion-channel inactivation from macroscopic current traces constitutes a classic biophysics problem in this class (see [4, 18, 31] for a review on the topic). Many of these molecular mechanisms were identified in the eighties and the nineties for different types of ion channels through single-channel electrophysiology recordings, fluctuation analysis, and analysis of gating currents [6, 10–12, 32]. However, analogous problems emerge in a great number of biological systems for which it is hard to design experiments to tease molecular schemes apart, or single-molecule experimental techniques are simply not yet available.

**Fig 5:**
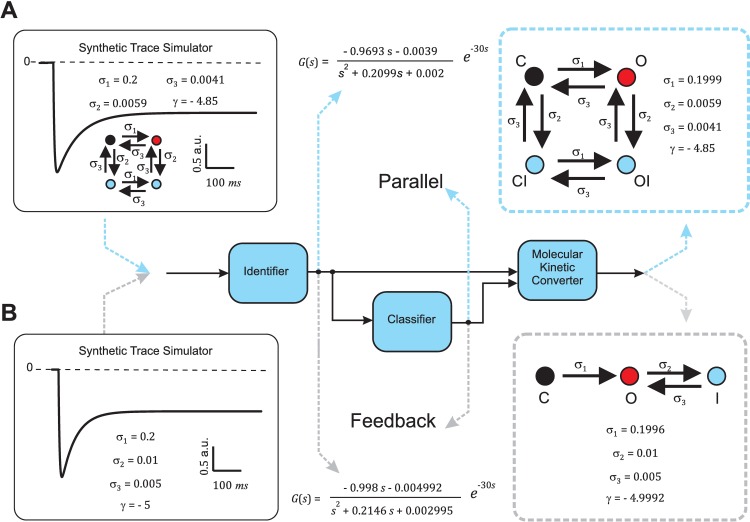
Similar traces from different molecular kinetic schemes. Application of SYSMOLE to two similar synthetic traces in response to an ideal step at *t* = 30 ms. The top trace (**A**) was generated with the Synthetic Trace Simulator (see *Results*section) by combining in parallel two processes with parameters *k*_*a*_ = - 5, *k*_*b*_ = 3, *τ*_*a*_ = 5, ms and *τ*_*b*_ = 100 ms. The correct molecular kinetic scheme was derived by SYSMOLE and the kinetic parameters obtained. The bottom trace (**B**) was also generated with the Synthetic Trace Simulator by combining in feedback two processes with parameters *k*_*a*_ = - 5, *k*_*b*_ = 2, *τ*_*a*_ = 5 ms and *τ*_*b*_ = 200 ms. The correct molecular kinetic scheme was derived by SYSMOLE and the kinetic parameters obtained. Boundary conditions used in the Classifier Module are *τ*_*a*_ ∈ [1, 9] ms, *τ*_*b*_ ∈ [50, 250] ms *k*_*a*_ ∈ [−20, 20], and *k*_*b*_ ∈ [−20, 20] for the parallel problem, and *k*_*b*_ ∈ [0, 20] for the feedback problem since combinations in feedback with *k*_*b*_ < 0 are unstable.

First we programmed an additional external module, the Synthetic Trace Simulator. This module generates a trace simulating each of the five molecular kinetic schemes associated with each configuration (Fig 4): first-order, second-order cascade, second-order feedback, second-order parallel addition, and second-order parallel subtraction. For practical purposes, in our implementation each molecular kinetic scheme is characterized by the parameters of two first-order processes *a* and *b* (*k*_*a*_, *k*_*b*_, *τ*_*a*_ and *τ*_*b*_) and the kinetic parameters *σ*_*k*_ and *γ* derived from the equations detailed in the previous section. As a proof of concept, we used the Synthetic Trace Simulator to simulate voltage-clamp experiments and generate, in response to a step in voltage, the macroscopic current trace for molecular kinetic schemes that include first-order processes describing the activation and inactivation of ion channels (Fig 5). Emulating the classic biophysics problem, we compared two extremely similar macroscopic traces arising from different molecular kinetic schemes (compare left panels on Fig 5A and 5B). The first simulated current trace (Fig 5A) emerged from a molecular scheme in which inactivation affected both the closed and the open states of the channel (Fig 4D, black: closed state, blue: inactivated states, red: open state). The trace was generated with activation (*a*) and inactivation (*b*) processes defined by parameters *k*_*a*_ = -5, *k*_*b*_ = 2, *τ*_*a*_ = 5 ms, and *τ*_*b*_ = 200 ms. The second simulated current trace was generated with a molecular kinetic scheme where inactivation only acted on the open state (Fig 4C, black: closed state, red: open state, blue: inactivated state). This trace was obtained with a molecular kinetic scheme associated with first-order activation (*a*) and inactivation (*b*) processes with parameters *k*_*a*_ = -5, *k*_*b*_ = 3, *τ*_*a*_ = 5 ms, and *τ*_*b*_ = 100 ms. We then analyzed both traces with SYSMOLE: first we identified the dynamics of the macroscopic trace elicited by the voltage step and summarized them in a transfer function *G*(*s*), then we classified *G*(*s*) to obtain the underlying configuration, and finally we obtained the corresponding molecular kinetic scheme using the MKC Module (Fig 5).

Our results indicate that our methodology can accurately retrieve the molecular kinetic scheme of these two similar traces. Analogous tests with less similar traces were also performed with successful results. In order to control for a possible bias associated with using the same environments for simulation and analysis, two different implementations of the Synthetic Trace Simulator were tested with practically identical results (see *Materials and Methods*).

### SYSMOLE is robust to noise in the macroscopic trace

Our previous results had concluded that the system-identification approach presented in the first part of the Results section (SYSMOLE) could be successfully applied to accurately elucidate the molecular kinetic scheme underlying a given macroscopic trace. However, these studies had been performed in the absence of noise, an unrealistic situation in any biological experimental setting. We decided to test the effects of noise on the performance of SYSMOLE.

**Fig 6:**
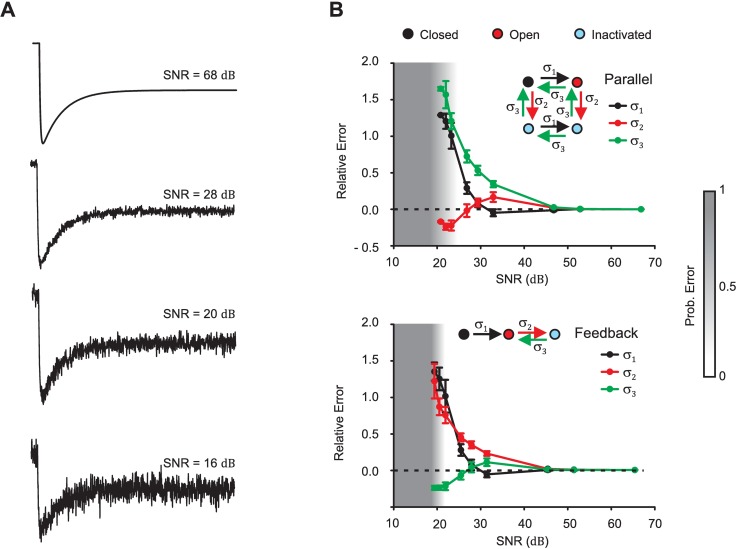
Effect of noise on kinetic parameters and probability of error in classification. (**A**) Representative traces of output signal *y*(*t*) used to test the effects of noise. Traces were generated with the Synthetic Trace Module (see *Results*section) combining two first-order processes with parameters *k*_*a*_ = - 5, *k*_*b*_ = 3, *τ*_*a*_ = 5 ms and *τ*_*b*_ = 100 ms in parallel with different levels of white Gaussian noise added. (**B**) Relative error in transition-rate kinetic parameters (*σ*_1_, *σ*_2_, and *σ*_3_) and probability of error in detection for parallel and feedback configurations. Each point in the graph depicts the mean and standard deviation of 100 simulations. Boundary conditions used in the Classifier Module are *τ*_*a*_ ∈ [1, 9] ms, *τ*_*b*_ ∈ [50, 250] ms, *k*_*a*_ ∈ [−20, 20], and *k*_*b*_ ∈ [−20, 20] for the parallel problem, and *k*_*b*_ ∈ [0, 20] for the feedback problem since combinations in feedback with *k*_*b*_ < 0 are unstable.

The robustness to noise of SYSMOLE will strongly depend on the features of the signals of interest and the type of noise existing on the trace. A useful approach to establish the reliability of SYSMOLE in the presence of noise is to simulate the types of experimental traces we are studying and test SYSMOLE when different levels of noise are added. We studied the effects of noise on the kinetic parameter values of the molecular kinetic scheme and the probability of error in classification of traces in which we observe a fast-activating process followed by a slower inactivation at the macroscopic level (Fig 5). These traces are representative of the types of traces obtained in voltage-clamp experiments with L-type calcium channels, and in the GPCR-heteromer signaling measurements presented in subsequent sections. By inspection of the traces (Figs 5 and 6A) one can easily conclude that the two processes are not combined in cascade, since this would be incompatible with a signal that reverses direction in the *y* axis (see Fig 2). Therefore, we focused our analysis on how noise affects the distinction between the feedback and parallel configurations, which based on our preliminary tests, is more prone to error due to the presence of noise in the trace. Noise tests were performed for all configurations and a wide range of combinations of first order parameters for the activation and inactivation processes with similar results.

Specifically, with the aid of the Synthetic Trace Simulator we generated synthetic traces involving two first-order processes characterized by parameters *τ*_*a*_ = 5 ms, *τ*_*b*_ = 100 ms, *k*_*a*_ = −5, and *k*_*b*_ = 3, using the molecular kinetic scheme associated with either the parallel subtraction (Fig 4E) or the feedback (Fig 4C) configurations. We generated multiple traces for each molecular kinetic scheme, to which we added increasing levels of white Gaussian noise (*N* = 100 simulations per level of noise). We analyzed each trace with SYSMOLE and measured (i) the relative error of the transition rate parameter values from the molecular kinetic schemes *σ*_1_, *σ*_2_ and *σ*_3_ (Fig 4D and 4E), and (ii) the probability of error in classification (*P*_*e*_) defined as the ratio between the number of correct classifications and the total number of simulations at a given level of noise (Fig 6B). It should be noted that the scaling factor *γ* is an adjustable parameter that shapes the amplitude of the signal and its sensitivity to noise has less relevance than the transition rates or the configuration.

In our analysis, traces arising from molecular kinetic schemes associated with the parallel and feedback configurations had similar robustness to noise (Fig 6B). The relative errors in the transition rate kinetic parameters began to degrade at a signal-to-noise ratio (SNR) of 47 dB, but stayed within a relative error of 0.5 at an SNR = 30 dB and above (*see Materials and Methods*section for a description of how the SNR was calculated). The probability of error in classification increased rapidly for traces with a SNR lower than 25 dB for the parallel configuration and 22 dB for the feedback configuration, reaching a *P*_*e*_ = 1 at 18 dB for both configurations. It is worth noting that the *P*_*e*_ represents not only the instances in which a trace in feedback configuration is mistakenly classified as a parallel configuration or vice versa, but also errors in which the trace is classified as arising from a higher-order (≥2) or first-order system. These two types of errors quickly dominate the probability of error in detection at high levels of noise as the SNR degrades.

Together, our results indicate that for the traces of interest one can perform a noise analysis with similar synthetic traces and establish for a given SNR the expected probability of error in classification and relative error in the transition-rate parameters of the molecular kinetic scheme. For traces of the types obtained in our experimental systems (L-type calcium channels and GPCR Gi and Gq), our results indicated that a SNR of 25 dB or better should provide the right identification of the molecular kinetic scheme and a low relative error in the kinetic parameters. Analogous studies revealed that a similar robustness to noise of SYSMOLE can be achieved in the presence of Brownian noise, and that the effect of noise on *P*_*e*_ can be improved through pre-processing by filtering the signal prior to application of SYSMOLE (S1 Text sections 4.1 and 4.2). We also established the potential applicability of SYSMOLE to recognizing gene-regulatory mechanisms associated with a feedback or parallel configurations in the presence of cell-to-cell variability in gene induction noise (S1 Text section 4.3).

### Analysis with SYSMOLE of L-type calcium channel experimental traces retrieves the mechanism of action of nifedipine

We decided to apply SYSMOLE to study the molecular mechanism of voltage-dependent inactivation of L-type calcium channels and the mechanism of action of nifedipine, a calcium channel blocker widely used clinically as an antianginal and antihypertensive drug [33, 34]. The inactivation mechanism of L-type channels has been extensively studied, and two major inactivation molecular mechanisms have been identified and characterized: a fast calcium-dependent inactivation mediated by the calcium ions entering inside the cells upon opening of the channel, and a voltage-dependent inactivation mediated by the voltage sensor located in the principal subunit and its movement upon depolarization to block the channel (see [35] for a review). Nifedipine, as part of the dihydropyridine class of channel blockers, binds to specific regions of the principal subunit to block the channel. The affinities of nifedipine for these binding regions differ depending on the state of the channel: nifedipine has a high affinity site for the inactivated state, a low affinity site accessible in the open state, and virtually no affinity sites for the closed state of the channel [36–39]. We tested whether we could derive molecular kinetic schemes consistent with the known molecular mechanism of voltage-dependent inactivation and nifedipine blockade of the L-type channels from experimental macroscopic current traces with the help of SYSMOLE.

**Fig 7:**
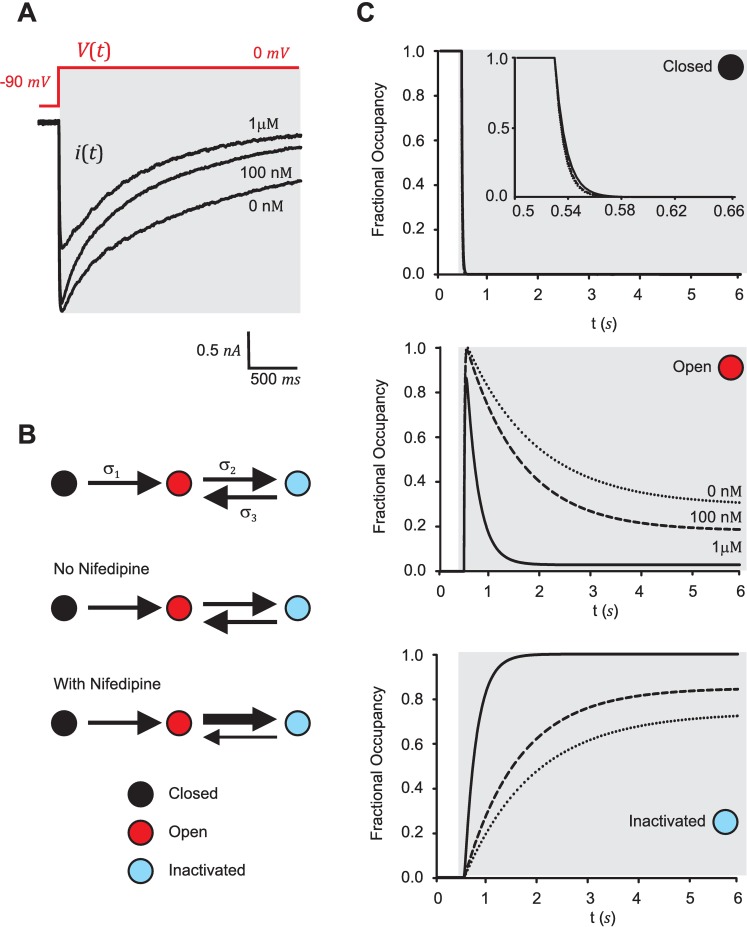
Molecular kinetic scheme and effects of nifedipine on L-type calcium channel traces. (**A**) Representative L-type calcium ion-channel current (output) elicited by voltage steps from -90 to 0 mV (input), first in the absence of nifedipine, and after addition of nifedipine at 100 nM and 1 μM concentrations. (**B**) Molecular kinetic scheme obtained by applying SYSMOLE to the traces and expected effects of nifedipine on transition rates. (**C**) Fractional occupancies as a function of time of the closed-channel (black), open-channel (red), and inactivated-channel (blue) states resulting from simulating the molecular kinetic scheme. Simulations were generated with the mean value of the kinetic parameters (N = 3–6 traces per treatment). Kinetic parameter values can be found on Table 1.

We used macroscopic current traces from voltage clamp (VC) experiments performed in the accessory radula closer muscle of *Aplysia californica* previously obtained by our group [40]. Currents through these channels were isolated from potassium currents by adding selective blockers (see *Materials and Methods*) and obtained in response to a depolarization step from - 90 mV to 0 mV. Calcium ions were replaced by barium in the solutions to remove the contribution of calcium-dependent inactivation and isolate the voltage-dependent inactivation of the channels. The currents were measured in the absence or presence of nifedipine (100 nM and 1 *μ*M) (see Fig 7A for representative current traces). Following the noise analysis described in the previous section, the SNR of all traces (> 38 dB for all cases) predicted a low probability of error in the identification by SYSMOLE of the correct molecular kinetic scheme as well as a low relative error for the transition rate parameters *σ*_*k*_ (Fig 6 and Table 1). In VC experiments the voltage is held constant while the current flowing through the ion channels in the cell membrane is measured based on the amount of current that an amplifier needs to supply to maintain the set voltage. We used the voltage as a function of time *V*(*t*) (a step from - 90 to 0 mV) as the input trace, and the recorded current *i*(*t*) as the output trace for SYSMOLE.

**Table 1:** Kinetic Parameters Ion-channel Traces.

[Nifedipine]	SNR(dB)	Config.	*σ*_1_	*σ*_2_	*σ*_3_	*γ*
0 nM	47.30 ± 5.92	f (100%)	0.48 ± 0.01	3x10^-3^± 7.95x10^-4^	1.18x10^-3^± 2.94x10^-4^	-2.41 ± 0.14
100 nM	51.98 ± 0.89	f (100%)	0.45 ± 0.03	4.40x10^-3^± 2.73x10^-4^	9.32x10^-4^± 2.35x10^-4^	-2.14 ± 0.05
1 μM	42.82 ± 3.57	f (100%)	0.39 ± 0.07	2.35x10^-2^± 2.5x10^-2^	6.76x10^-4^± 8.73x10^-4^	-2.56 ± 1.18

N = 3–8 traces per group. f (100%) indicates feedback configuration was obtained for 100% of the traces.

In biological terms, we can explain the current trace *i*(*t*) elicited by a step in voltage in a VC experiment using a molecular kinetic scheme as follows. Initially, the system is at steady-state and the fractional occupancies (i.e. the fraction of channels in any particular state) for each of the states are constant and the population of channels in dynamic equilibrium. At this point in time, the current readout is also constant (basal current) and proportional to the fractional occupancy in the open state. When the input steps to a higher voltage, the rate constants which govern the transitions between states change and the population of channels redistributes accordingly among the states. This redistribution is accompanied by a transient in the fractional occupancies, and thus in the current recorded. At the end of this transient a new steady-state level of current is reached. The value of the rate constants characterizing the transitions before the step in voltage are unknown, and therefore, the *σ*_*k*_ strictly represent the change in rate associated with the input, in this case a step in voltage.

The results of applying SYSMOLE to derive molecular kinetic schemes from macroscopic traces of L-type calcium channel currents obtained in the voltage-clamp (VC) experiments described, consistently yielded the molecular kinetic scheme associated with a feedback configuration (Fig 7B and Table 1). In the absence of nifedipine, inactivation of the channel is due to the voltage-dependent inactivation, which is engaged when the channel opens due to the depolarization of the membrane. As such, a molecular kinetic scheme in which the channels need to open to inactivate is consistent with this molecular mechanism (Fig 7B) [41]. Based on the known mechanism of action of nifedipine, and due to the high affinity binding of nifedipine to the inactivated state, we expected the presence of this drug to reduce the transition rate from the inactivated state to the open state (I→O). Similarly, due to the existence of a binding site for nifedipine in the open state of the channel, we also expected an increase in the transition rate between the open state and the inactivated state (O→I), which now includes two different states the one reached through voltage-dependent inactivation, and the inactivation due to the presence of nifedipine. Finally, we expected nifedipine not to affect the transition between the closed and the open state (C→O) since it does not bind to the channel in the resting closed state. All these effects were extracted by SYSMOLE as detailed in Table 1, and illustrated by the temporal evolution of the fractional occupancies in the obtained molecular kinetic scheme (Fig 7C).

Together, our results showed that SYSMOLE performed strongly with experimental traces and allowed us to derive the correct molecular kinetic scheme of the voltage-dependent inactivation of the L-type calcium channels and to predict the molecular mechanism of action of nifedipine.

### SYSMOLE can be applied to differentiate between *cis-* and *trans*-activation in heteromeric G protein-coupled receptor complexes

G protein-coupled receptors (GPCRs) are membrane-bound receptors that transduce extracellular binding of molecules into intracellular signals by activating a class of heterotrimeric proteins called G proteins [42, 43]. Each subclass of G protein is associated with a signaling pathway with specific actions inside the cell in response to stimuli [44]. Classically, the signaling paradigm was considered to follow the rule that one ligand binds to one receptor which activates only one pathway. However, extensive biochemical and biophysical evidence has revealed the existence of GPCR homo- and hetero-dimers/oligomers that differentially alter G protein signaling. Furthermore, the regulation of these complexes is found to play a critical role in normal physiology and disease (see [45] for a review). Despite their importance, the molecular signaling mechanisms of GPCR heteromeric and homomeric complexes remain largely unknown.

The dysregulation of the heteromeric complex formed by the Gi-coupled metabotropic glutamate receptor 2 (mGluR2) and the Gq-coupled serotonin 2A receptor (5-HT2AR) has been linked to schizophrenia [46]. Preclinical and clinical studies suggest that activation of mGluR2 in the complex by allosteric and orthosteric agonists could represent a new therapeutic approach to treat schizophrenia and other disorders [47–49]. Our group has published work showing that psychedelic drugs upset the G_i_/G_q_ signaling balance associated with the mGluR2/5-HT2AR complex by reducing G_i_ and increasing G_q_ signaling, while antipsychotic drugs restore the natural G_i_/G_q_ balance. This is achieved through a crosstalk mechanism in which a ligand acting on one of the receptors, either 5-HT2AR or mGluR2, can change the signaling on their counterpart receptor [50]. However, the mechanism by which this crosstalk is achieved molecularly through the mGluR2/5-HT2AR complex remains to be elucidated.

Two possible molecular mechanisms occurring through cross-conformational changes have been postulated to explain the crosstalk observed between mGluR2 and 5-HT2AR through the mGluR2/5-HT2AR complex. One referred to as *cis-*activation, in which the ligand-free receptor is not able to activate G proteins unless the first one signals and is bound to G proteins. The second one referred to as *trans-*activation, in which the signal from the ligand-bound receptor is transmitted to the neighboring receptor which then signals by activating G proteins.

**Fig 8:**
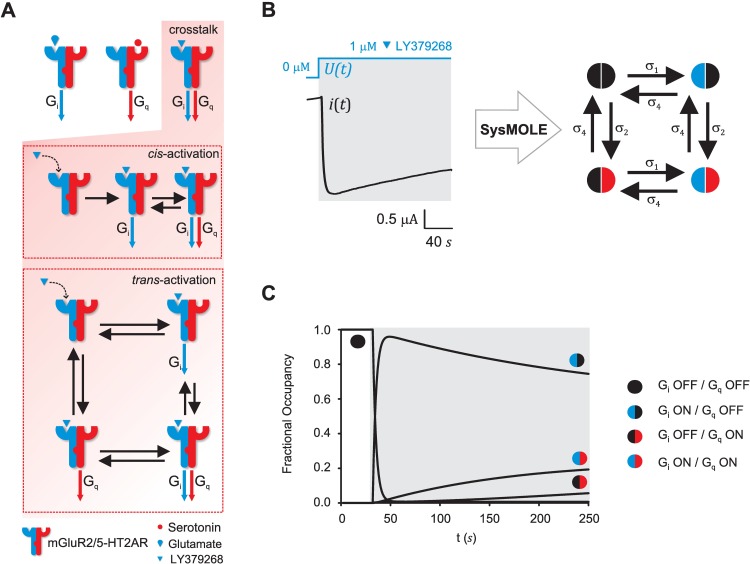
Molecular kinetic scheme of crosstalk signaling in the mGluR2/5-HT2AR heteromeric complex in response to glutamate antipsychotics. (**A**) (Top) Scheme of crosstalk signaling through the mGluR2/5-HT2AR complex. In response to the endogenous ligands glutamate and serotonin, the mGluR2/5-HT2AR complex signals through G_i_ and G_q_ signaling pathways respectively. Certain ligands such as LY379268, a dominant mGluR2 agonist with antipsychotic properties, are able to crosstalk through the complex and activate both G_i_ and G_q_. (Bottom) Molecular kinetic schemes associated with the *cis*-activation and the *trans*-activation theories of crosstalk. (**B**) (Left) Representative G-protein sensitive ion-channel (GIRK4*) current trace (output) elicited by a step in LY379268 concentration from 0 to 1 μM (input). (Right) Molecular kinetic scheme obtained by applying SYSMOLE to the traces. (**C**) Fractional occupancies as a function of time of the four states in the molecular kinetic scheme: G_i_ OFF / G_q_ OFF, G_i_ ON / G_q_ OFF, G_i_ ON / G_q_ ON, and G_i_ OFF / G_q_ ON. The molecular kinetic scheme postulates a new state G_i_ OFF / G_q_ ON and supports the *trans*-activation theory for crosstalk through the complex (see *Results*section). Simulations were generated with the mean value of the kinetic parameters (N = 5 traces). Kinetic parameter values can be found on Table 2.

The *cis-*activation and *trans-*activation crosstalk theories could be distinguished in terms of molecular kinetic schemes (Fig 8A). We decided to apply SYSMOLE to derive the molecular kinetic scheme from macroscopic traces obtained from mGluR2/5-HT2AR in response to LY379268, a ligand belonging to the promising new class of glutamate antipsychotics [51], which has been shown to elicit a strong crosstalk between the mGluR2 and the 5-HT2AR [46, 50]. While glutamate and serotonin, the two endogenous neurotransmitters, activate G_i_ and G_q_ pathways respectively through the mGluR2/5-HT2AR complex, LY379268 can activate both G_i_ and G_q_ despite only binding mGluR2 (Fig 8A).

Ion channels have been extensively used to measure GPCR signaling activity. For our study, we expressed the mGluR2 and 5-HT2AR receptors together with a G protein sensitive potassium ion channel (GIRK4*) in *Xenopus* oocytes and measured using two-electrode voltage clamp the current flowing through the channel as a function of time (see *Materials and Methods*). The input signal for SYSMOLE was a step in concentration of LY379268, and the output signal the macroscopic current through the GIRK4* channel. Since G_i_ signaling increases current through the channel (downward direction) and Gq signaling decreases the current through the channel (upward direction) [52] the traces obtained (Fig 8B) were perfectly suited to apply SYSMOLE to understand the relationship between G_i_ and G_q_ signaling and derive a molecular kinetic scheme. It should also be noted that the noise level in these traces results in a SNR clearly below the cut-off for error-free detection (Fig 6). Should the traces be noisier or have a type of noise added other than Gaussian, prior to analyzing the experimental traces SYSMOLE should be tested with synthetic traces and its robustness to noise for that type of trace determined (see S1 Text sections 4.1 and 4.2 for a description of the robustness of SYSMOLE to Brownian noise, and pre-processing strategies to increase the SNR in the trace).

Given that both the opening and closing of the reporter channel are faster processes (milliseconds) compared to G_i_ and G_q_ activation (seconds) the trace does not capture them, and the resulting molecular kinetic scheme allows us to distinguish states resulting from combinations in which the G_i_ is OFF (no change in current) or ON (current through the channel is increasing), G_q_ is OFF (no change in current) or ON (current through the channel is decreasing) or combinations.

Application of SYSMOLE to different macroscopic traces obtained in response to LY379268 (Table 2) yielded the molecular kinetic scheme associated with the parallel subtraction (Fig 8B right) with four states, which can be related to different signaling states depending on whether the G_i_ or G_q_ signaling is ON or OFF in the complex. Additional controls were performed with injection of only one receptor (mGluR2 or 5-HT2AR) and endogenous ligands to ensure that the traces captured the crosstalk effects through mGluR2/5-HT2AR. From the molecular kinetic scheme, it can be inferred that G_i_ and G_q_ signaling are processes that occur in parallel upon LY379268 binding to mGluR2, and that activation of G_q_ does not require G_i_ activation. A close look at the fractional occupancy in each state as a function of time reveals that due to the strong G_i_ signal activation of mGluR2 by LY379268 (dominant agonist [50]), most of the complex molecules move from the G_i_ OFF/ G_q_ OFF state to G_i_ ON/ G_q_ OFF state and then transition directly to the G_i_ ON/ G_q_ ON state. Interestingly, there is also a fraction of the molecules that transition from the original G_i_ OFF / G_q_ OFF state directly to G_i_ OFF/ G_q_ ON state, a molecular state that had not been postulated before. This fraction, albeit small, amounts to approximately one third of the total fractional occupancy in the G_i_ ON/G_q_ ON state when the system reaches steady state Our results support the hypothesis of a *trans-*activation as the mechanism of crosstalk of the mGluR2/5-HT2AR heteromeric complex. It should be noted that the assignment of these ON and OFF states does not come directly from SYSMOLE, which is agnostic to the biological interpretation and is here used to generate hypotheses. The molecular existence of these states would need to be explicitly tested in further experiments (see *Discussion*).

**Table 2:** Kinetic Parameters mGluR2/5-HT2AR Complex Traces.

SNR(dB)	Config.	*σ*_1_	*σ*_2_	*σ*_3_	*γ*
50.4 ± 3.81	p (100%)	0.30 ± 0.04	1.73x10^-3^± 4.96x10^-4^	2.73x10^-3^± 1.21x10^-3^	-2.29 ± 0.73

N = 5 traces. p (100%) indicates parallel configuration was obtained for 100% of the traces.

Together, these results exemplify the usefulness of the SYSMOLE system-identification analytical tool in studying heteromeric signaling.

## Discussion

A correct characterization of the dynamics of different molecular processes in the cell is fundamental for understanding normal physiological and pathophysiological responses. In most biological systems the direct study of these microscopic molecular processes is not yet possible due to experimental limitations, and only overall macroscopic cell responses to stimuli (macroscopic traces) can be obtained. Here we introduced a method that combines computational and analytical approaches to extract information from the macroscopic trace regarding the molecular microscopic processes and the way in which they are combined. This method, SYSMOLE, conveniently describes the dynamics of these microscopic processes in the form of a molecular kinetic scheme analogous to those used in biophysics and pharmacology.

SYSMOLE is implemented in modular fashion. We first detailed the mathematical underpinnings of each of the three modules that are included in SYSMOLE: the Identifier Module, the Classifier Module, and the Molecular Kinetic Converter Module. Through rigorous and systematic evaluations, we explored SYSMOLE’s limitations and robustness to noise (see also S1 Text section 4). We validated the performance of SYSMOLE on experimental traces by correctly identifying the molecular kinetic scheme describing the activation and inactivation of L-type calcium channels, as well as the mechanism of action of nifedipine, a calcium channel blocker clinically used in patients with cardiovascular disease. Furthermore, we applied SYSMOLE to study the signaling crosstalk mechanism observed in the heteromeric complex formed by mGluR2 and 5-HT2AR in response to LY379268, a compound that belongs to the new class of glutamate antipsychotics [53]. A Matlab toolbox with all the functions that we have developed is available in Matlab Central (https://www.mathworks.com/matlabcentral/fileexchange/61465-sysmole). The SYSMOLE toolbox contains an implementation of the modules for second-order systems and third-order systems with three poles and two zeros. It contains the experimental data used in the manuscript, and also provides the user with templates to simulate and test noise models and apply SYSMOLE to traces from their biological system of interest.

In the broader context of systems biology, other powerful methods exist to extract network models from biological data in different domains (see reviews [54] and [55] for examples in allosteric networks and functional genomics, respectively). As one could expect, each method and approach has its strengths and is most adapted to deal with specific aspects or features of the data and the biological system. A large group of methods obtain possible networks from scarce and unhomogeneously-sampled measurements or traces [54–60]. SYSMOLE tackles a different set of questions and is best adapted to capture subtle features in adequately-sampled macroscopic traces that underlie essential differences in the biological system. As such, SYSMOLE is a complementary approach to other methods, and as the experimental techniques evolve to enable sampling more often and acquiring more data per unit time, tools like SYSMOLE will become particularly useful to have in the computational biologist toolbox.

Compared to other methods that deal with adequately-sampled data, SYSMOLE is a flexible method that can be generalized to the study of multiple biological systems and applied to different types of biological traces. Various methods have been developed to derive molecular kinetic schemes from macroscopic current traces in the ion channel biophysics field. The best results have been obtained utilizing maximum likelihood methods [19, 20] and covariance methods [61]. These methods strongly rely on underlying models to characterize ion channel current flow which, following decades of biophysics research, are usually available [4]. Unfortunately, these methods cannot be easily generalized to most biological systems, for which reliable models of the molecular microscopic processes are typically unavailable and large numbers of measurements are impractical. Furthermore, many of the available methods are not able to tackle stimuli (inputs) with different dynamics, a key feature of many biological systems and physiological responses. SYSMOLE overcomes these limitations. It does not rely on underlying models, it extracts information per input-output trace pair, and it incorporates information about the dynamics of the stimulus. Utilizing a system identification step based on a transfer function allows SYSMOLE to capture the dynamics without relying on a physical model. By extending the concept of a Markov chain and linking one of the states to the input, SYSMOLE can extract the molecular kinetic scheme from macroscopic traces elicited by inputs with various dynamics. Finally, the flexible properties of SYSMOLE have the added benefit to retrospectively allow leveraging of important biological information from the vast repository of macroscopic traces collected through the years by different labs.

The overall performance of SYSMOLE is determined by the module with the lowest performance, which in turn depends on the characteristics of each particular macroscopic trace and physiological process under study. The performance of the ARX (autoregressive) method used in the Identifier Module will degrade as the noise increases, or as the sampling frequency decreases and the input and output traces become insufficiently sampled in time. Based on our experience these limitations can be overcome through the application of classical preprocessing and filtering techniques to the traces [22] (see S1 Text section 4.1) together with an acquisition sampling frequency of $\frac{1}{2T}$, with *T* being the time constant of the fastest process that we wish to capture. The Classifier Module will not perform properly if information regarding the differences in time constants of the processes under study is unavailable. Lacking this information will result in an improperly bound optimization problem which will not exclude the parameter region where the feedback and parallel solutions coincide (S1 Text section 1). This might hinder the applicability of the method to processes characterized by extremely similar time constants. Finally, the Molecular Kinetic Converter is an analytical conversion, and thus in principle will always perform correctly. To confirm the validity of the conversion for every case, internal checks are included along the mathematical derivation to ensure the rules presented are sound (S1 Text section 2).

For didactic simplicity, we focused in our presentation on a second-order system, in which two first-order processes are combined in different arrangements. However, each module in SYSMOLE can be scaled to multiple processes. The ARX implementation of the Identifier module can easily extract up to 6 to 10 processes and does not represent a bottleneck for scalability [24, 25]. The series of comparisons implemented in the Classifier Module can be updated to incorporate additional combinations of numbers of poles and zeros. The optimization problems needed to differentiate these additional combinations are also easily scalable to higher-order systems, and thus to multiple processes. Finally, the Molecular Kinetic Scheme can be scaled using a modular approach in which each combination of two processes is considered as a module, and the connection of that module to another module or process also occurs through three possible canonical configurations (see the *Scalibility* section in S1 Text for a description of the application of SYSMOLE to third-order systems with three poles and two zeros).

Application of SYSMOLE to the study of crosstalk through the mGluR2/5-HT2AR heteromeric receptor complex in *Xenopus* oocytes illustrates its strength in generating new hypotheses regarding molecular mechanisms. Our results indicate that LY379268, a glutamate antipsychotic that binds to mGluR2, can signal through G_q_ in the absence of any ligand bound to 5-HT2AR, which suggests *trans*-activation as the molecular mechanism of crosstalk. This analysis postulated the existence of a signaling state in which the receptor signals through G_q_ without signaling through Gi. This new state might have important implications in understanding the pharmacology of new glutamate antipsychotic compounds [53, 62, 63]. Since SYSMOLE strictly obtains a Markov-chain state model, the assignment of each of the states to a particular signaling conformation (Gi ON or OFF, Gq ON or OFF) is also postulated. Confirmation of the existence of these states will require further experiments in which it is possible to assume that the occupancies of the states of one or more states is constant or altered. One could for example perform similar crosstalk experiments (Fig 8) with complexes in which either of the receptors that form the heteromer are mutated such that it cannot bind Gi or Gq. Furthermore, relevance of this new postulated signaling state in neurons should also be assessed.

In conclusion, despite the extensive efforts devoted to the development single-molecule experimental techniques, alternatives are needed for the analysis of macroscopic traces. SYSMOLE, a hybrid computational and analytical tool designed to analyze macroscopic traces and derive molecular kinetic schemes, enables the user to extract information regarding the microscopic processes involved in the response to a given stimulus from the available macroscopic traces. SYSMOLE has also the added benefit to be able to leverage important biological information from the vast repository of macroscopic traces to generate new hypotheses.

## Materials and methods

### Identifier module

The Identifier Module was implemented in Matlab (www.mathworks.com) using the System Identification Toolbox ARX functions. Details on loss-function minimization strategies in prediction-error-identifications can be found in [25].

### Classifier module

The parallel and feedback optimization problems described in the main text were solved using a trust-region-reflective algorithm [64] included in the Optimization Toolbox of Matlab (www.mathworks.com).

### Molecular kinetic converter

Details on the derivation of the molecular kinetic schemes for each canonical configuration can be found in the supporting materials (S1 Text).

### Synthetic trace simulator

Two implementations were used: one in Matlab, the environment in which SYSMOLE is implemented, and one using COPASI [65], a software application for simulation and analysis of biochemical networks and their dynamics.

### Signal-to-noise ratio calculation

In order to obtain the signal-to-noise ratio (SNR) one needs to calculate the average power of the signal *y*(*t*) *P*_*Y*_, as well as the noise power. The signal’s average power can be directly calculated in the time domain as:

$$P_{Y}=\frac{1}{N}\sum_{n=0}^{\infty }{y[n]}^{2}$$ (47)

where *y*[*n*] is the sequence resulting from discretizing *y*(*t*) with sample period *T*_*s*_ and sample number *n* = 1 … *N*.

The noise power depends on the noise characteristics and the bandwidth of *y*(*t*), which varies depending on the parameters of the first order processes that give rise to the signal (*k*_*a*_, *k*_*b*_, *τ*_*a*_, and *τ*_*b*_). All the necessary information needed to calculate this value is provided by the Identifier Module. On the one hand, in the Identifier Module we had included disturbances in the form of white Gaussian noise with variance *λ* filtered and added to the output (see Identifier). On the other hand, this module yields the transfer function *G*(*s*) that will allow us to calculate the bandwidth of the signal *BW*. In order to test whether a noise characterization as WGN is adequate for the type of experimental traces we analyzed, we confirmed that the *λ* value estimated by the Identifier Module matched the value of the variance of *y*(*t*) before stimulation both for the simulated synthetic traces as for the experimental traces. Once *λ* and *BW* are obtained, the noise power *P*_*N*_ filtered to the signal’s bandwidth can be calculated as

$$P_{N}=\int_{-\infty }^{\infty }{\left|G(f)\right|}^{2}S_{n}(f)df=\int_{-\infty }^{\infty }{\left|G(f)\right|}^{2}\lambda \;df$$ (48)

where *G*(*f*) is the frequency response of the transfer function *G*(*s*), and *S*_*n*_(*f*) is the power spectral density of the noise. Assuming an ideal low-pass filter, the value of this integral can be approximated to

$$P_{N}≃2\lambda .BW$$ (49)

For our analysis we used Eq (49) with a bandwidth calculated at 3 dB drop in gain. A design of a non-ideal low-pass filter with cutoff frequency equal to *BW*, 1 dB ripple in the pass band, and 60 dB attenuation in the stop band yielded practically identical results.

### Calcium-channel voltage-clamp traces

Digitized current and voltage values were obtained from previous voltage-clamp experiments performed in the accessory radula closer muscle of *Aplysia californica*. Solutions, drug delivery, and experimental conditions are described in detail in reference [66].

### G-protein coupled receptor activity measurements in *Xenopus* oocytes

Oocytes were isolated and microinjected with equal volumes (50 nl), as previously described [67]. In all two-electrode voltage-clamp experiments (TEVC), oocytes were injected with 1 ng of mGluR2, 2 ng of 5-HT2AR, and 2 ng of GIRK4*, and were maintained at 18 ºC for 14 days before recording.

Whole-cell currents were measured by conventional two-electrode voltage-clamp (TEVC) with a GeneClamp 500 amplifier (Axon Instruments, Union City, CA, USA. A high-potassium (HK) solution was used to superfuse oocytes (96 mM KCl, 1 mM NaCl, 1 mM MgCl, 5 mM KOH/HEPES, pH 7.4) to obtain a reversal potential for potassium (*E*_*k*_) close to zero. Inwardly rectifying potassium currents through GIRK4* were obtained by clamping the cells at - 80 mV. A solution of 3 mM of BaCl in HK solution was perfused at the end of each trace to ensure that the current measured corresponded to GIRK4*, as previously described [52].

## Supporting information

S1 Fig Signature of poles, zeros, and gain. Second-order transfer-function values of the time constants associated with the poles and zero, and the gain resulting from applying the three canonical configurations to combine two first-order processes defined by time constants *τ*_*a*_ = 5 ms and *τ*_*b*_ = 80 ms and strengths (gains), *k*_*a*_ and *k*_*b*_, ranging from -10 to 10.
(TIF)

S2 Fig Convergence of the Classifier Module. Study of the convergence properties of the parallel and feedback optimization problems when tested with two processes, *a* and *b*, characterized by *τ*_*a*_ = 15 ms and *τ*_*b*_ = 150 ms combined in parallel (**A**) with *k*_*a*_ ∈ [−10, 10] and *k*_*b*_ ∈ [−10, 10], or in feedback (**B**) with *k*_*a*_ ∈ [−10, 10], and *k*_*b*_ ∈ [0, 10] (negative *k*_*b*_ yields unstable positive-feedback systems). Left panel indicates, in red the value for the cost function of the parallel problem (*f*_*valp*_) and in blue the value for the cost function of the feedback problem (*f*_*valf*_), as a function of the number of iterations in the optimization algorithm. First column indicates the normalized Euclidean distance between the real parameter values (*τ*_*a*_, *τ*_*b*_, *k*_*a*_, *k*_*b*_) and the solution obtained by the parallel optimization problem (top), and the feedback optimization problem (bottom). Second column indicates the values for the cost functions of the parallel optimization problem *f*_*valp*_ (top) and the feedback optimization problem *f*_*valf*_ (bottom). Finally, the third column indicates the value of the logical condition tested by the Classifier Module to discriminate between parallel and the feedback configurations (Fig 3). Identical studies were performed testing the Classifier Module with all possible configurations for *τ*_*a*_ = [5, 10, 15, 20] and *τ*_*b*_ = [100, 150, 200, 300] with similar results. Boundary conditions used for optimization problems are *k*_*a*_ and *k*_*b*_ ∈ [−20, 20], *τ*_*a*_ ∈ [1, 50] ms and *τ*_*b*_ ∈ [50, 350] ms. We restricted *k*_*b*_ ∈ [0, 20] for the feedback problem since negative *k*_*b*_ would yield positive-feedback unstable systems for negative values. The results indicate that the parallel and feedback optimization problems converge for a wide range of parameters and that the implementation of the Classifier Module is capable of discerning between the parallel and feedback configurations in second-order transfer functions.
(TIF)

S1 Text Additional mathematical derivations and scalability. (1) Constraints for optimization problems. (2) Derivation of molecular kinetic schemes for the canonical configurations. (3) Scalability. (4) Noise.
(DOCX)

S1 Data Experimental Traces. (1) L-type calcium ion-channel current elicited by voltage steps from -90 to 0 mV, first in the absence of nifedipine, and after addition of nifedipine at 100 nM and 1 μM concentration. (2) G-protein sensitive ion-channel (GIRK4*) current traces elicited by a step in LY379268 concentration from 0 to 1 μM.
(ZIP)

## References

[1] Marks F, Klingmüller U, Müller-Decker K. (2009) Cellular signal processing. an introduction to the molecular mechanims of signal transduction New York: Garland Science, Taylor and Francis Group, LLC.

[2] Bray D. (1995) Protein molecules as computational elements in living cells. Nature 376: 307–312. 10.1038/376307a0 7630396

[3] Barabasi AL, Oltvai ZN. (2004) Network biology: Understanding the cell's functional organization. Nat Rev Genet 5: 101–113. 10.1038/nrg1272 14735121

[4] Hille B. (2001) Ionic channels of excitable membranes, 3rd ed. Sunderland, MA: Sinauer Associates.

[5] Sigg D. (2014) Modeling ion channels: Past, present, and future. J Gen Physiol 144: 7–26. 10.1085/jgp.201311130 24935742 PMC4076515

[6] Colquhoun D, Hawkes AG. (1981) On the stochastic properties of single ion channels. Proc R Soc Lond B Biol Sci 211: 205–235. 6111797 10.1098/rspb.1981.0003

[7] Gration KA, Lambert JJ, Ramsey RL, Rand RP, Usherwood PN. (1982) Closure of membrane channels gated by glutamate receptors may be a two-step process. Nature 295: 599–603. 6120466 10.1038/295599a0

[8] Cull-Candy SG, Usowicz MM. (1987) Multiple-conductance channels activated by excitatory amino acids in cerebellar neurons. Nature 325: 525–528. 10.1038/325525a0 2433594

[9] Neher E, Sakmann B. (1992) The patch clamp technique. Sci Am 266: 44–51. 1374932 10.1038/scientificamerican0392-44

[10] Hamill OP, Marty A, Neher E, Sakmann B, Sigworth FJ. (1981) Improved patch-clamp techniques for high-resolution current recording from cells and cell-free membrane patches. Pflugers Arch 391: 85–100. 6270629 10.1007/BF00656997

[11] Qin F, Auerbach A, Sachs F. (1996) Estimating single-channel kinetic parameters from idealized patch-clamp data containing missed events. Biophys J 70: 264–280. S0006-3495(96)79568-1 [pii]. 10.1016/S0006-3495(96)79568-1 8770203 PMC1224925

[12] Qin F, Auerbach A, Sachs F. (2000) A direct optimization approach to hidden markov modeling for single channel kinetics. Biophys J 79: 1915–1927. S0006-3495(00)76441-1 [pii]. 10.1016/S0006-3495(00)76441-1 11023897 PMC1301083

[13] Ritort F. (2006) Single-molecule experiments in biological physics: Methods and applications. J Phys Condens Matter 18: R531–83. 10.1088/0953-8984/18/32/R01 21690856

[14] Roy R, Hohng S, Ha T. (2008) A practical guide to single-molecule FRET. Nat Methods 5: 507–516. 10.1038/nmeth.1208 18511918 PMC3769523

[15] Hartwell LH, Hopfield JJ, Leibler S, Murray AW. (1999) From molecular to modular cell biology. Nature 402: C47–52. 10.1038/35011540 10591225

[16] Jordan JD, Landau EM, Iyengar R. (2000) Signaling networks: The origins of cellular multitasking. Cell 103: 193–200. S0092-8674(00)00112-4 [pii]. 11057893 10.1016/s0092-8674(00)00112-4 PMC3619409

[17] Bray D. (2003) Molecular networks: The top-down view. Science 301: 1864–1865. 10.1126/science.1089118 14512614

[18] Kienker P. (1989) Equivalence of aggregated markov models of ion-channel gating. Proc R Soc Lond B Biol Sci 236: 269–309. 2471201 10.1098/rspb.1989.0024

[19] Milescu LS, Yamanishi T, Ptak K, Mogri MZ, Smith JC. (2008) Real-time kinetic modeling of voltage-gated ion channels using dynamic clamp. Biophys J 95: 66–87. 10.1529/biophysj.107.118190 18375511 PMC2426646

[20] Milescu LS, Akk G, Sachs F. (2005) Maximum likelihood estimation of ion channel kinetics from macroscopic currents. Biophys J 88: 2494–2515. S0006-3495(05)73305-1 [pii]. 10.1529/biophysj.104.053256 15681642 PMC1305347

[21] Oppenheim AV, Willsky AS, Hamid-Nawab S. (1999) Signals and systems. 2nd ed. Upper Saddle River, NJ: Prentice Hall.

[22] Godfrey KR. (1993) Perturbation signals for system identification. London, UK: Prentice Hall.

[23] Tan AH, Godfrey KR. (2009) A guide to the design and selection of perturbation signals. Proeceeedings 48th IEEE Conference on Decision and Control: 464–469.

[24] Söderström T, Fan H, Carlsson B, Bigi S. (1997) Least squares parameter estimation of continuous-time ARX models from discrete-time data. IEEE Transaction on Automatic Control 42, NO.5.

[25] Ljung L. (1999) System identification. theory for the user. 2nd ed.: Prentice Hall.

[26] Oppenheim AV, Schafer RW. (1989) Discrete-time signal processing: Prentice hall.

[27] Mei C. (2002) On teaching the simplification of block diagrams. Int J of Engng Ed 18: 697–703.

[28] Karayanakis NM. (1995) Advanced system modeling and simulation with block diagram languages. Boca Raton, Florida, US: CRC Press. 350 p.

[29] Song L, Magleby KL. (1994) Testing for microscopic reversibility in the gating of maxi K+ channels using two-dimensional dwell-time distributions. Biophys J 67: 91–104. S0006-3495(94)80458-8 [pii]. 10.1016/S0006-3495(94)80458-8 7919030 PMC1225338

[30] Rothberg BS, Magleby KL. (2001) Testing for detailed balance (microscopic reversibility in ion channel gating. Biophys J 80: 3025–3026. S0006-3495(01)76268-6 [pii]. 10.1016/S0006-3495(01)76268-6 11432375 PMC1301486

[31] Patlak J. (1991) Molecular kinetics of voltage-dependent na+ channels. Physiol Rev 71: 1047–1080. 1656476 10.1152/physrev.1991.71.4.1047

[32] Colquhoun D, Sakmann B. (1985) Fast events in single-channel currents activated by acetylcholine and its analogues at the frog muscle end-plate. J Physiol 369: 501–557. 2419552 10.1113/jphysiol.1985.sp015912 PMC1192660

[33] Go AS, Bauman MA, Coleman King SM, Fonarow GC, Lawrence W, et al (2014) An effective approach to high blood pressure control: A science advisory from the american heart association, the american college of cardiology, and the centers for disease control and prevention. Hypertension 63: 878–885. 10.1161/HYP.0000000000000003 24243703 PMC10280688

[34] Yancy CW, Jessup M, Bozkurt B, Butler J, Casey DE Jr, et al (2013) 2013 ACCF/AHA guideline for the management of heart failure: Executive summary: A report of the american college of cardiology Foundation/American heart association task force on practice guidelines. Circulation 128: 1810–1852. 10.1161/CIR.0b013e31829e8807 23741057

[35] Catterall WA. (2000) Structure and regulation of voltage-gated Ca2+ channels. Annu Rev Cell Dev Biol 16: 521–555. 10.1146/annurev.cellbio.16.1.521 11031246

[36] Bean BP. (1984) Nitrendipine block of cardiac calcium channels: High-affinity binding to the inactivated state. Proc Natl Acad Sci U S A 81: 6388–6392. 6093100 10.1073/pnas.81.20.6388 PMC391929

[37] Gurney AM, Nerbonne JM, Lester HA. (1985) Photoinduced removal of nifedipine reveals mechanisms of calcium antagonist action on single heart cells. J Gen Physiol 86: 353–379. 2414392 10.1085/jgp.86.3.353 PMC2228798

[38] Schuster A, Lacinova L, Klugbauer N, Ito H, Birnbaumer L, et al (1996) The IVS6 segment of the L-type calcium channel is critical for the action of dihydropyridines and phenylalkylamines. Embo j 15: 2365–2370. 8665843 PMC450166

[39] Lacinova L, An RH, Xia J, Ito H, Klugbauer N, et al (1999) Distinctions in the molecular determinants of charged and neutral dihydropyridine block of L-type calcium channels. J Pharmacol Exp Ther 289: 1472–1479. 10336541

[40] Brezina V, Evans CG, Weiss KR. (1994) Enhancement of ca current in the accessory radula closer muscle of aplysia californica by neuromodulators that potentiate its contractions. J Neurosci 14: 4393–4411. 7913122 10.1523/JNEUROSCI.14-07-04393.1994 PMC6577045

[41] Catterall WA. (1995) Structure and function of voltage-gated ion channels. Annu Rev Biochem 64: 493–531. 10.1146/annurev.bi.64.070195.002425 7574491

[42] Rosenbaum DM, Rasmussen SG, Kobilka BK. (2009) The structure and function of G-protein-coupled receptors. Nature 459: 356–363. 10.1038/nature08144 19458711 PMC3967846

[43] Audet M, Bouvier M. (2012) Restructuring G-protein- coupled receptor activation. Cell 151: 14–23. 10.1016/j.cell.2012.09.003 23021212

[44] Oldham WM, Hamm HE. (2008) Heterotrimeric G protein activation by G-protein-coupled receptors. Nat Rev Mol Cell Biol 9: 60–71. nrm2299 [pii]. 10.1038/nrm2299 18043707

[45] Gonzalez-Maeso J. (2011) GPCR oligomers in pharmacology and signaling. Mol Brain 4: 20-6606-4-20. 10.1186/1756-6606-4-20 PMC3128055 21619615

[46] Gonzalez-Maeso J, Ang RL, Yuen T, Chan P, Weisstaub NV, et al (2008) Identification of a serotonin/glutamate receptor complex implicated in psychosis. Nature 452: 93–97. 10.1038/nature06612 18297054 PMC2743172

[47] Moreno JL, Holloway T, Albizu L, Sealfon SC, Gonzalez-Maeso J. (2011) Metabotropic glutamate mGlu2 receptor is necessary for the pharmacological and behavioral effects induced by hallucinogenic 5-HT2A receptor agonists. Neurosci Lett 493: 76–79. 10.1016/j.neulet.2011.01.046 21276828 PMC3064746

[48] Patil ST, Zhang L, Martenyi F, Lowe SL, Jackson KA, et al (2007) Activation of mGlu2/3 receptors as a new approach to treat schizophrenia: A randomized phase 2 clinical trial. Nat Med 13: 1102–1107. 10.1038/nm1632 17767166

[49] Kinon BJ, Zhang L, Millen BA, Osuntokun OO, Williams JE, et al (2011) A multicenter, inpatient, phase 2, double-blind, placebo-controlled dose-ranging study of LY2140023 monohydrate in patients with DSM-IV schizophrenia. J Clin Psychopharmacol 31: 349–355. 10.1097/JCP.0b013e318218dcd5 21508856

[50] Fribourg M, Moreno JL, Holloway T, Provasi D, Baki L, et al (2011) Decoding the signaling of a GPCR heteromeric complex reveals a unifying mechanism of action of antipsychotic drugs. Cell 147: 1011–1023. 10.1016/j.cell.2011.09.055 22118459 PMC3255795

[51] Moreno JL, Sealfon SC, Gonzalez-Maeso J. (2009) Group II metabotropic glutamate receptors and schizophrenia. Cell Mol Life Sci 66: 3777–3785. 10.1007/s00018-009-0130-3 19707855 PMC2792875

[52] Hatcher-Solis C, Fribourg M, Spyridaki K, Younkin J, Ellaithy A, et al (2014) G protein-coupled receptor signaling to kir channels in xenopus oocytes. Curr Pharm Biotechnol 15: 987–995. 25374032 10.2174/1389201015666141031111916 PMC4426293

[53] Ellaithy A, Younkin J, Gonzalez-Maeso J, Logothetis DE. (2015) Positive allosteric modulators of metabotropic glutamate 2 receptors in schizophrenia treatment. Trends Neurosci 38: 506–516. 10.1016/j.tins.2015.06.002 26148747 PMC4530036

[54] Gerek ZN, Ozkan SB. (2011) Change in allosteric network affects binding affinities of PDZ domains: Analysis through perturbation response scanning. PLoS Comput Biol 7: e1002154 10.1371/journal.pcbi.1002154 21998559 PMC3188487

[55] Braga-Neto UM, Marques ET Jr. (2006) From functional genomics to functional immunomics: New challenges, old problems, big rewards. PLoS Comput Biol 2: e81 10.1371/journal.pcbi.0020081 16863395 PMC1523295

[56] Bonneau R, Reiss DJ, Shannon P, Facciotti M, Hood L, et al (2006) The inferelator: An algorithm for learning parsimonious regulatory networks from systems-biology data sets de novo. Genome Biol 7: R36 gb-2006-7-5-r36 [pii]. 10.1186/gb-2006-7-5-r36 16686963 PMC1779511

[57] Greenfield A, Hafemeister C, Bonneau R. (2013) Robust data-driven incorporation of prior knowledge into the inference of dynamic regulatory networks. Bioinformatics 29: 1060–1067. 10.1093/bioinformatics/btt099 23525069 PMC3624811

[58] Thorne T, Stumpf MP. (2012) Inference of temporally varying bayesian networks. Bioinformatics 28: 3298–3305. 10.1093/bioinformatics/bts614 23074260 PMC3519458

[59] Babtie AC, Kirk P, Stumpf MP. (2014) Topological sensitivity analysis for systems biology. Proc Natl Acad Sci U S A 111: 18507–18512. 10.1073/pnas.1414026112 25512544 PMC4284538

[60] Oates CJ, Dondelinger F, Bayani N, Korkola J, Gray JW, et al (2014) Causal network inference using biochemical kinetics. Bioinformatics 30: i468–74. 10.1093/bioinformatics/btu452 25161235 PMC4147905

[61] Celentano JJ, Hawkes AG. (2004) Use of the covariance matrix in directly fitting kinetic parameters: Application to GABAA receptors. Biophys J 87: 276–294. 10.1529/biophysj.103.036632 15240464 PMC1304350

[62] Baki L, Fribourg M, Younkin J, Eltit JM, Moreno JL, et al (2016) Cross-signaling in metabotropic glutamate 2 and serotonin 2A receptor heteromers in mammalian cells. Pflugers Arch 468: 775–793. 10.1007/s00424-015-1780-7 26780666 PMC4842341

[63] Moreno JL, Miranda-Azpiazu P, Garcia-Bea A, Younkin J, Cui M, et al (2016) Allosteric signaling through an mGlu2 and 5-HT2A heteromeric receptor complex and its potential contribution to schizophrenia. Sci Signal 9: ra5 10.1126/scisignal.aab0467 26758213 PMC4819166

[64] Dennis J, Vicente L. (1996) Trust-region interior-point algorithms for minimization problems with simple bounds. Applied Mathematics and Parallel Computing.

[65] Hoops S, Sahle S, Gauges R, Lee C, Pahle J, et al (2006) Copasi—a complex pathway simulator. Bioinformatics 22:10.1093/bioinformatics/btl485 17032683

[66] Brezina V, Evans CG, Weiss KR. (1994) Characterization of the membrane ion currents of a model molluscan muscle, the accessory radula closer muscle of aplysia californica. III. depolarization-activated ca current. J Neurophysiol 71: 2126–2138. 7931506 10.1152/jn.1994.71.6.2126

[67] Lopes CM, Zhang H, Rohacs T, Jin T, Yang J, et al (2002) Alterations in conserved kir channel-PIP2 interactions underlie channelopathies. Neuron 34: 933–944. 12086641 10.1016/s0896-6273(02)00725-0

