# Correction: Elucidation of molecular kinetic schemes from macroscopic traces using system identification

**DOI:** 10.1371/journal.pcbi.1006539

**Published:** 2018-10-16

**Authors:** Miguel Fribourg, Diomedes E. Logothetis, Javier González-Maeso, Stuart C. Sealfon, Belén Galocha-Iragüen, Fernando Las-Heras Andrés, Vladimir Brezina

There is an error in the affiliation for the first author. Miguel Fribourg is also affiliated with the Department of Signals Systems and Radiocommunications, Universidad Politécnica de Madrid, Madrid, Spain.
