# Supplementary figures and images for: Elucidation of molecular kinetic schemes from macroscopic traces using system identification
Source: PLoS Comput Biol. 2017 Feb 13;13(2):e1005376. doi: 10.1371/journal.pcbi.1005376 (PMC5330533; doi:10.1371/journal.pcbi.1005376)

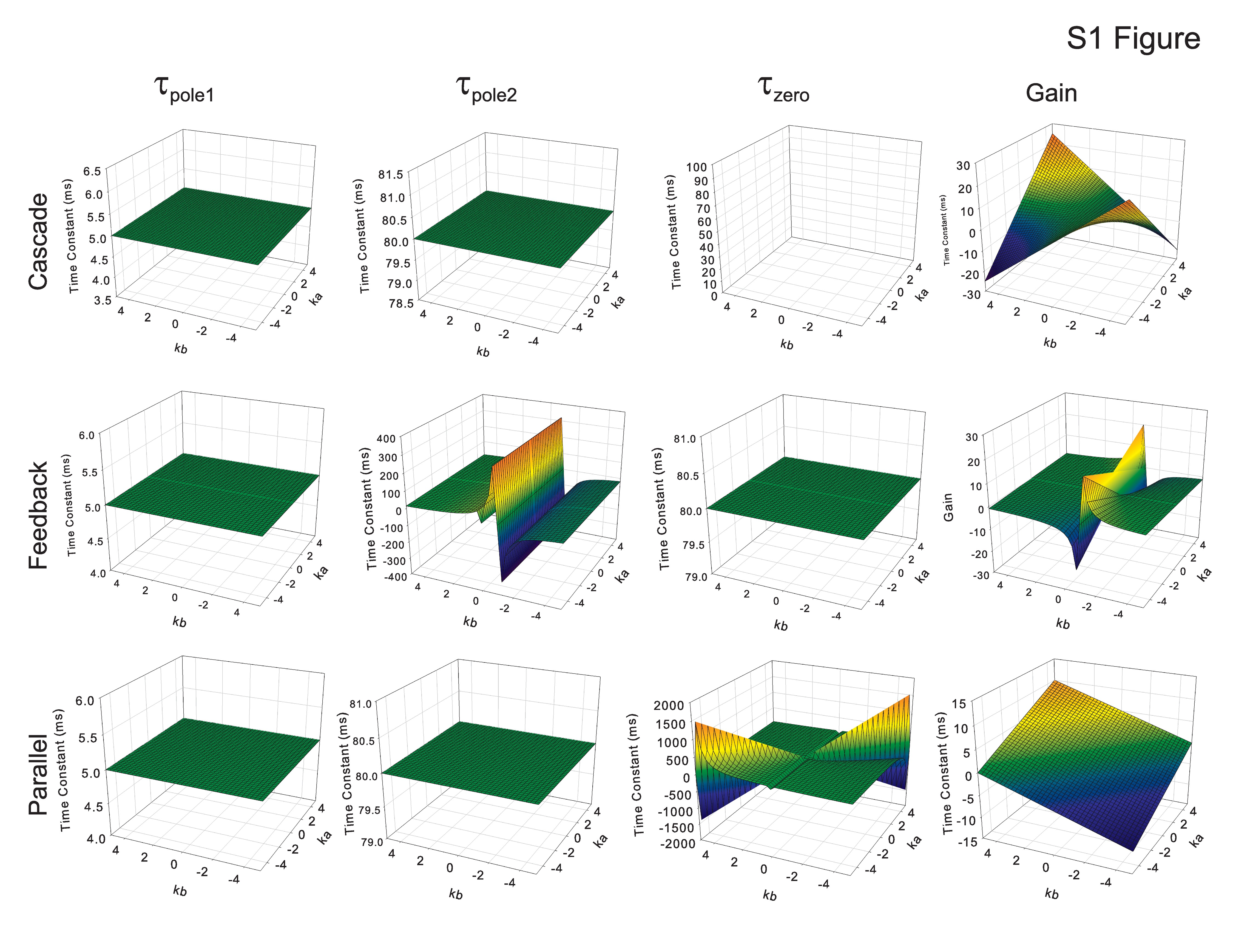

Supplement: S1 Fig — Second-order transfer-function values of the time constants associated with the poles and zero, and the gain resulting from applying the three canonical configurations to combine two first-order processes defined by time constants τa = 5 ms and τb = 80 ms and strengths (gains), ka and kb, ranging from -10 to 10. (TIF) [file pcbi.1005376.s001.tif]

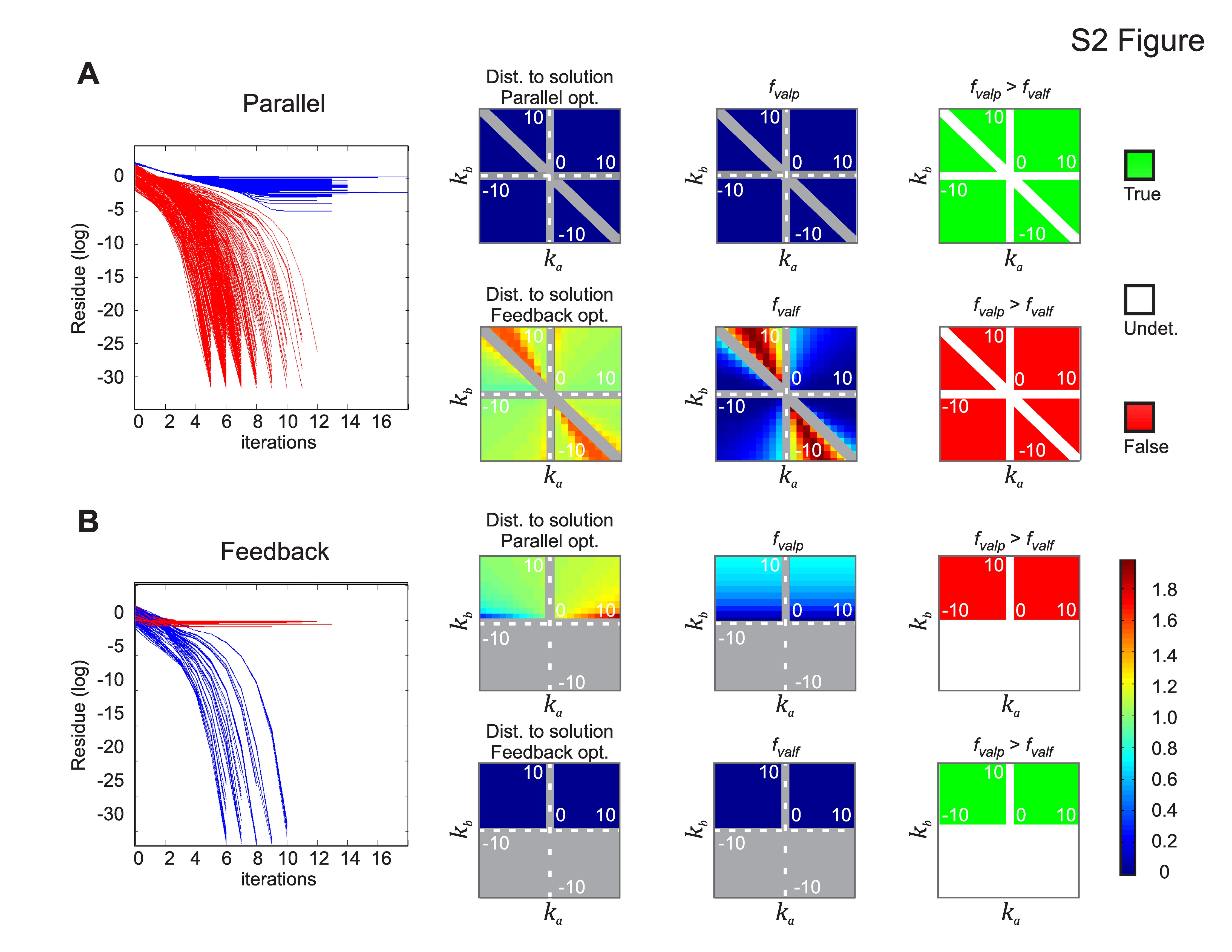

Supplement: S2 Fig — Study of the convergence properties of the parallel and feedback optimization problems when tested with two processes, a and b, characterized by τa = 15 ms and τb = 150 ms combined in parallel (A) with ka ∈ [−10, 10] and kb ∈ [−10, 10], or in feedback (B) with ka ∈ [−10, 10], and kb ∈ [0, 10] (negative kb yields unstable positive-feedback systems). Left panel indicates, in red the value for the cost function of the parallel problem (fvalp) and in blue the value for the cost function of the feedback problem (fvalf), as a function of the number of iterations in the optimization algorithm. First column indicates the normalized Euclidean distance between the real parameter values (τa, τb, ka, kb) and the solution obtained by the parallel optimization problem (top), and the feedback optimization problem (bottom). Second column indicates the values for the cost functions of the parallel optimization problem fvalp (top) and the feedback optimization problem fvalf (bottom). Finally, the third column indicates the value of the logical condition tested by the Classifier Module to discriminate between parallel and the feedback configurations (Fig 3). Identical studies were performed testing the Classifier Module with all possible configurations for τa = [5, 10, 15, 20] and τb = [100, 150, 200, 300] with similar results. Boundary conditions used for optimization problems are ka and kb ∈ [−20, 20], τa ∈ [1, 50] ms and τb ∈ [50, 350] ms. We restricted kb ∈ [0, 20] for the feedback problem since negative kb would yield positive-feedback unstable systems for negative values. The results indicate that the parallel and feedback optimization problems converge for a wide range of parameters and that the implementation of the Classifier Module is capable of discerning between the parallel and feedback configurations in second-order transfer functions. (TIF) [file pcbi.1005376.s002.tif]
