# Supplementary material for: Elucidation of molecular kinetic schemes from macroscopic traces using system identification
Source: PLoS Comput Biol. 2017 Feb 13;13(2):e1005376. doi: 10.1371/journal.pcbi.1005376 (PMC5330533; doi:10.1371/journal.pcbi.1005376)
Supplement: S1 Text — (1) Constraints for optimization problems. (2) Derivation of molecular kinetic schemes for the canonical configurations. (3) Scalability. (4) Noise. (DOCX) [file pcbi.1005376.s003.docx]

Supporting Text

Text S1 | Additional Mathematical Derivations and Scalability

**1. Constraints for Optimization Problems**

The Classifier Module distinguishes the parallel and feedback second-order configurations by solving two optimization problems with constraints on the parameters that characterize the first-order systems $G_{a}$ and $G_{b}$ that give rise to the second-order system. These parameters are $b_{a}$, $b_{b}$, $\omega_{a}$ and $\omega_{b}$ (see main text) and the constraints defined as $\omega_{amin}\leq\omega_{a}\leq\omega_{amax}$ , $\omega_{bmin}\leq\omega_{b}\leq\omega_{bmax}$, $b_{amin}\leq b_{a}\leq b_{amax}$ , and $b_{bmin}\leq b_{b}\leq b_{bmax}$. The choice of these constraints is guided by prior information available regarding the two biological processes of interest, e.g. knowing that one process is one order of magnitude faster than the second process. More formally, one can derive a set of equations that define the region of the parameter space that should be avoided in order to ensure accurate classification as follows. The transfer function resulting from the combination in parallel of two first-order systems $G_{ap=}\frac{b_{ap}}{s+\omega_{ap}}$ and $G_{bp=}\frac{b_{bp}}{s+\omega_{bp}}$ can be expressed as:

| $G_{p}(s)=\frac{\left( b_{ap}+b_{bp} \right)s+b_{ap}\omega_{bp}+b_{bp}\omega_{ap}}{(s+\omega_{ap})(s+\omega_{bp})}$ | (1) |
| --- | --- |

Analogously, the transfer function resulting from the combination in feedback of two first-order systems $G_{af=}\frac{b_{af}}{s+\omega_{af}}$ and $G_{bf=}\frac{b_{bf}}{s+\omega_{bf}}$ can be expressed as:

| $G_{f}(s)=\frac{b_{af}{(s+\omega}_{bf})}{(s+\omega_{af})(s+\omega_{bf}+b_{bf})}$ | (2) |
| --- | --- |

By comparing each coefficient in the transfer function we can obtain the expressions for the set of parameters $b_{ap}$,$b_{bp}$,$b_{af}$,$b_{af}$,$\omega_{ap}$,$\omega_{bp}$,$\omega_{af}$, and $\omega_{bf}$ that would yield the same transfer function $(G_{p}\left( s \right)=G_{f}\left( s \right))$.

| $\left\{ \begin{matrix} \omega_{ap}=\omega_{af} \\ \omega_{bp}=\omega_{bf}+b_{bf} \\ \begin{matrix} b_{ap}=b_{af}-b_{bp} \\ b_{bp}=\frac{b_{af}\omega_{bf}+b_{ap}\omega_{bp}}{\omega_{ap}} \end{matrix} \end{matrix} \right.$ | (3) |
| --- | --- |

Consequently, the region of parameter space explored by the Classifier Module and defined by the constraints, should exclude values that when given to $b_{ap}$,$b_{bp}$,$b_{af}$,$b_{af}$,$\omega_{ap}$,$\omega_{bp}$,$\omega_{af}$, and $\omega_{bf}$ satisfy the equations above. In practice, one can find that this holds true when prior information about the time scales of the processes under study is included in the choice of the constraints, as stated in the main text.

**2. Derivation of Molecular Kinetic Schemes for the Canonical Configurations**

In the following subsections we present a comprehensive study and analytical derivation of the molecular kinetic schemes for first-order systems and the three possible canonical block-diagrams for second-order systems: cascade, feedback, and parallel. These mathematical derivations follow the steps described in the main text for the implementation of the Molecular Kinetic Converter Module.

**2.1 First-order System**

1. Number of states$=n+1=$ 2

2. We identify the nodes and include the transitions in the diagram (see Figure 2.1).

| 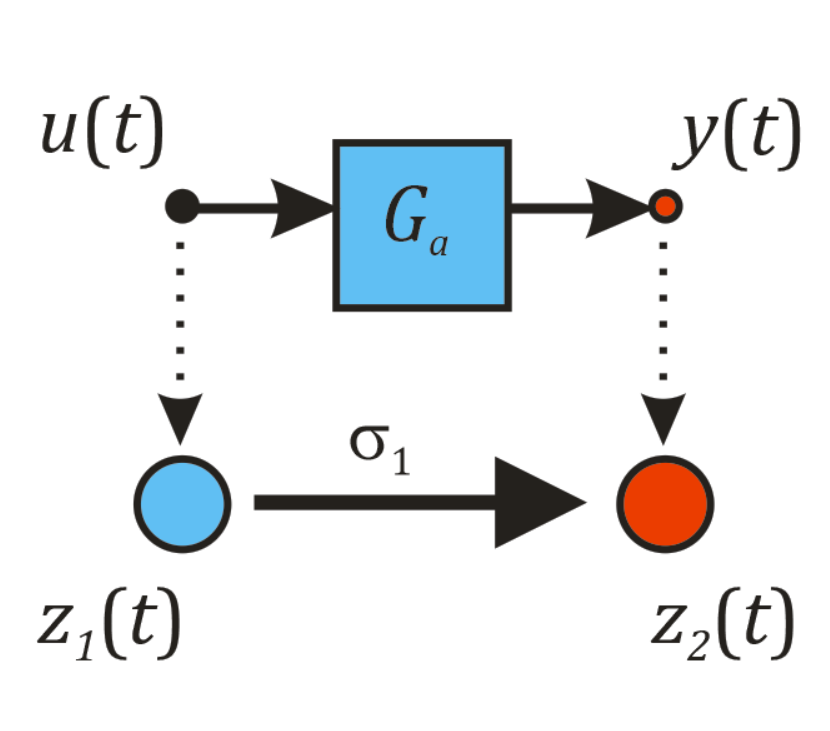 |
| --- |
| ***Figure 2.1.*** *First-order System.* |

3. We check that the principle of microscopic reversibility is satisfied.

4. We build the system of equations

| $\left\{ \begin{aligned} \frac{dz_{2}(t)}{dt}=\sigma_{1}z_{1}\left( t \right) \mathrm{ODE} Transition 1 \\ y\left( t \right)=\gamma z_{2}\left( t \right) Observable Equation \\ u\left( t \right)=z_{1}\left( t \right)+z_{2}\left( t \right) Mass Equation \end{aligned} \right.$ | (4) |
| --- | --- |

5. We Laplace transform the system assuming $z_{2}\left( 0 \right)=0$ for flexibility

| $\left\{ \begin{aligned} sZ_{2}(s)=\sigma_{1}Z_{1}\left( s \right) T1 \\ Y(s)=\gamma Z_{2}\left( s \right) O \\ U\left( s \right)=Z_{1}\left( s \right)+Z_{2}\left( s \right) M \end{aligned} \right.$ | (5) |
| --- | --- |

6. We obtain ${G(s)}_{kin}$by isolating $\frac{Y(s)}{U(s)}$ from equations T1, O and M.

| ${G(s)}_{kin}=\frac{Y(s)}{U(s)}=\frac{\sigma_{1}\gamma}{s+\sigma_{1}}$ | (6) |
| --- | --- |

7. From the block diagram we obtain $G(s)$

| $G(s)=\frac{b_{a}}{s+\omega_{a}}$ | (7) |
| --- | --- |

8. We compare the terms in ${G(s)}_{kin}$ and $G(s)$ and obtain the following equations

| $\sigma_{1}=\omega_{a}$ | (8) |
| --- | --- |
| $\left. {G(s)}_{kin} \right\rfloor_{s\to0}=\gamma=\left. G(s) \right\rfloor_{s\to0}=\frac{b_{a}}{\omega_{a}}\underset{\Rightarrow}{}\gamma=\frac{b_{a}}{\omega_{a}}$ | (9) |

**2.2 Second-order System: Cascade**

1. Number of states$=n+1=$ 3

2. We identify the nodes and include the transitions in the diagram (see Figure 2.2).

| 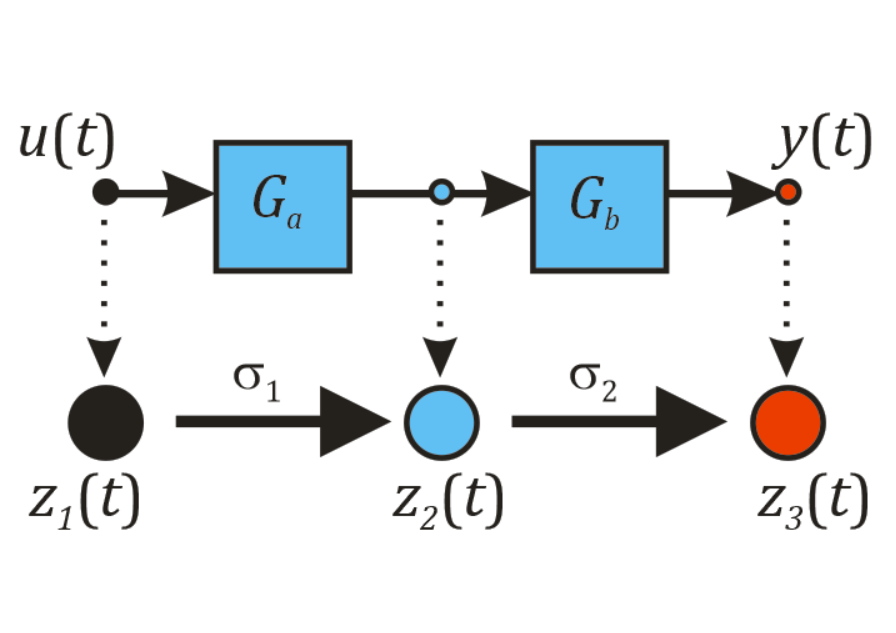 |
| --- |
| ***Figure 2.2.*** *Two first-order systems* $G_{a}$ *and* $G_{b}$ *connected in Cascade.* |

3. We check that the principle of microscopic reversibility is satisfied.

4. We build the system of equations

| $\left\{ \begin{matrix} \frac{dz_{3}(t)}{dt}=\sigma_{2}z_{2}\left( t \right) ODE Transition 1 \\ \frac{dz_{2}(t)}{dt}=\sigma_{1}z_{1}\left( t \right)-\sigma_{2}z_{2}\left( t \right) ODE Transition 2 \\ \begin{matrix} y\left( t \right)=\gamma z_{3}\left( t \right) Observable Equation \\ u\left( t \right)=z_{1}\left( t \right)+z_{2}\left( t \right)+z_{3}\left( t \right) Mass Equation \end{matrix} \end{matrix} \right.$ | (10) |
| --- | --- |
|  |  |

5. We Laplace transform the system assuming $z_{2}\left( 0 \right)=0$ and $z_{3}\left( 0 \right)=0$ for flexibility

| $\left\{ \begin{matrix} sZ_{3}(s)=\sigma_{2}Z_{2}\left( s \right) T1 \\ sZ_{2}(s)=\sigma_{1}Z_{1}\left( s \right)-\sigma_{2}Z_{2}\left( s \right) T2 \\ \begin{matrix} Y\left( s \right)=\gamma Z_{3}\left( s \right) O \\ U\left( s \right)=Z_{1}\left( s \right)+Z_{2}\left( s \right)+Z_{3}\left( s \right) M \end{matrix} \end{matrix} \right.$ | (11) |
| --- | --- |

6. We obtain ${G(s)}_{kin}$by isolating $\frac{Y(s)}{U(s)}$ from equations T1, T2, O and M.

| ${G(s)}_{kin}=\frac{Y(s)}{U(s)}=\frac{\gamma\sigma_{1}\sigma_{2}}{(s+\sigma_{1})(s+\sigma_{2})}$ | (12) |
| --- | --- |

7. From the block diagram we obtain $G(s)$

| $G(s)=\frac{b_{a}b_{b}}{\left( s+\omega_{a} \right)(s+\omega_{b})}$ | (13) |
| --- | --- |

8. We compare terms in in ${G(s)}_{kin}$ and $G(s)$ and obtain the following equations

| $\sigma_{1}=\omega_{a}$ | (14) |
| --- | --- |
| $\sigma_{2}=\omega_{b}$ | (15) |
| $\left. {G(s)}_{kin} \right\rfloor_{s\to0}=k_{a}k_{b}=\left. G(s) \right\rfloor_{s\to0}=\gamma\underset{\Rightarrow}{}\gamma=k_{a}k_{b}$ | (16) |

**2.3 Second-order System: Feedback**

1. Number of states$=n+1=$ 3

2. We identify the nodes and include the transitions in the diagram (see Figure 2.3). It should be noted that due to the subtraction operation present in the block diagram, the signal flows in opposite direction through $G_{a}$ and $G_{b}$ and this is reflected accordingly in the transitions depicted in the diagram.

| 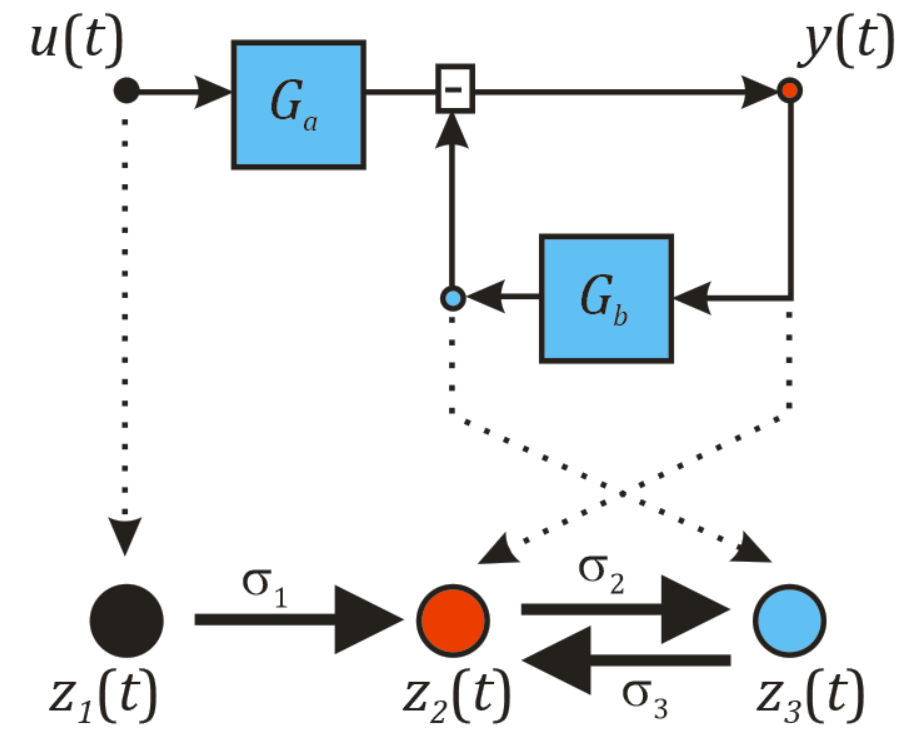 |
| --- |
| ***Figure 2.3.*** *Two first-order systems* $G_{a}$ *and* $G_{b}$ *connected in Feedback.* |

3. We check that the principle of microscopic reversibility is satisfied.

4. We build the system of equations

| $\left\{ \begin{matrix} \frac{dz_{3}(t)}{dt}=\sigma_{2}z_{2}\left( t \right)-\sigma_{3}z_{3}(t) ODE Transition 1 \\ \frac{dz_{2}(t)}{dt}=\sigma_{1}z_{1}\left( t \right)-\sigma_{2}z_{2}\left( t \right)+\sigma_{3}z_{3}(t) ODE Transition 2 \\ \begin{matrix} y\left( t \right)=\gamma z_{2}\left( t \right) Observable Equation \\ u\left( t \right)=z_{1}\left( t \right)+z_{2}\left( t \right)+z_{3}\left( t \right) Mass Equation \end{matrix} \end{matrix} \right.$ | (17) |
| --- | --- |

5. We Laplace transform the system assuming $z_{2}\left( 0 \right)=0$ and $z_{3}\left( 0 \right)=0$ for flexibility

| $\left\{ \begin{matrix} sZ_{3}\left( s \right)=\sigma_{2}Z_{2}\left( s \right)-\sigma_{3}Z_{3}(s) T1 \\ sZ_{2}\left( s \right)=\sigma_{1}Z_{1}\left( s \right)-\sigma_{2}Z_{2}\left( s \right)+\sigma_{3}Z_{3}(s) T2 \\ \begin{matrix} Y\left( s \right)=\gamma Z_{3}\left( s \right) O \\ U\left( s \right)=Z_{1}\left( s \right)+Z_{2}\left( s \right)+Z_{3}\left( s \right) M \end{matrix} \end{matrix} \right.$ | (18) |
| --- | --- |

6. We obtain ${G(s)}_{kin}$by isolating $\frac{Y(s)}{U(s)}$ from equations T1, T2, O and M.

| ${G(s)}_{kin}=\frac{Y(s)}{U(s)}=\frac{\gamma\sigma_{1}{(s+\sigma}_{3})}{(s+\sigma_{1})(s+\sigma_{3}+\sigma_{2})}$ | (19) |
| --- | --- |

7. From the block diagram we obtain $G(s)$

| $G(s)=\frac{b_{a}{(s+\omega}_{b})}{\left( s+\omega_{a} \right)(s+\omega_{b}+b_{b})}$ | (20) |
| --- | --- |

8. Comparing terms in ${G(s)}_{kin}$ and $G(s)$ we obtain the following equations

| $\sigma_{1}=\omega_{a}$ | (21) |
| --- | --- |
| $\sigma_{2}=b_{b}$ | (22) |
| $\sigma_{3}=\omega_{b}$ | (23) |
| $\left. {G(s)}_{kin} \right\rfloor_{s\to0}=\frac{\gamma\sigma_{1}\sigma_{3}}{\sigma_{1}(\sigma_{3}+\sigma_{2})}=\left. G(s) \right\rfloor_{s\to0}=\frac{\gamma\omega_{a}\omega_{b}}{\omega_{a}(\omega_{b}+b_{b})}\underset{\Rightarrow}{}\gamma=k_{a}$ | (24) |

**2.4 Second-order System: Parallel Addition**

1. Number of states$=n+1=$ 3

2. We identify the nodes and include the transitions in the diagram (see Figure 2.4).

| 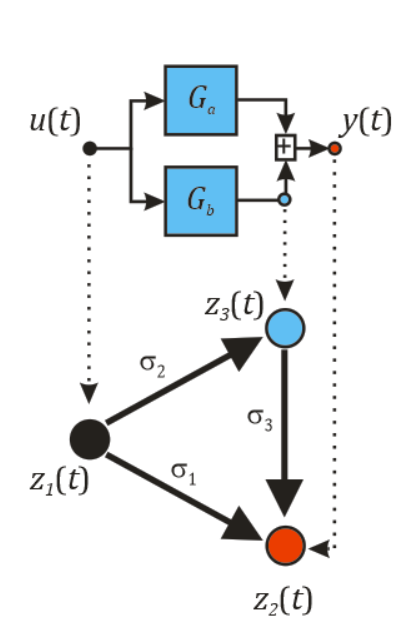 |
| --- |
| ***Figure 2.4.*** *Two first-order systems* $G_{a}$ *and* $G_{b}$ *added in Parallel.* |

3. We build the system of equations

| $\left\{ \begin{matrix} \frac{dz_{3}(t)}{dt}=\sigma_{2}z_{1}\left( t \right)-\sigma_{3}z_{3}(t) ODE Transition 1 \\ \frac{dz_{2}(t)}{dt}=\sigma_{1}z_{1}\left( t \right)+\sigma_{3}z_{3}\left( t \right) ODE Transition 2 \\ \begin{matrix} y\left( t \right)=\gamma z_{2}\left( t \right) Observable Equation \\ u\left( t \right)=z_{1}\left( t \right)+z_{2}\left( t \right)+z_{3}\left( t \right) Mass Equation \end{matrix} \end{matrix} \right.$ | (25) |
| --- | --- |

4. We Laplace transform the system assuming $z_{2}\left( 0 \right)=0$ and $z_{3}\left( 0 \right)=0$ for flexibility

| $\left\{ \begin{matrix} sZ_{3}\left( s \right)=\sigma_{2}Z_{1}\left( s \right)-\sigma_{3}Z_{3}(s) T1 \\ sZ_{2}\left( s \right)=\sigma_{1}Z_{1}\left( s \right)+\sigma_{3}Z_{3}(s) T2 \\ \begin{matrix} Y\left( s \right)=\gamma Z_{2}\left( s \right) O \\ U\left( s \right)=Z_{1}\left( s \right)+Z_{2}\left( s \right)+Z_{3}\left( s \right) M \end{matrix} \end{matrix} \right.$ | (26) |
| --- | --- |

5. We obtain ${G(s)}_{kin}$by isolating $\frac{Y(s)}{U(s)}$ from equations T1, T2, O and M.

| ${G(s)}_{kin}=\frac{Y(s)}{U(s)}=\frac{\gamma\left( s+\frac{\sigma_{1}\sigma_{3}+\sigma_{3}\sigma_{2}}{\sigma_{1}} \right)}{(s+\sigma_{1})(s+\sigma_{2}+\sigma_{3})}$ | (27) |
| --- | --- |

6. From the block diagram we obtain $G(s)$

| $G(s)=\frac{\left( b_{a}+b_{b} \right){s+b}_{a}\omega_{b}{+b}_{b}\omega_{a}}{\left( s+\omega_{a} \right)(s+\omega_{b})}$ | (28) |
| --- | --- |

7. Comparing terms in ${G(s)}_{kin}$ and $G(s)$ we obtain the following equations

| $\sigma_{1}=\frac{b_{a}+b_{b}}{k_{a}+k_{b}}$ | (29) |
| --- | --- |
| $\sigma_{2}=\omega_{b}-\sigma_{1}$ | (30) |
| $\sigma_{3}=\omega_{a}$ | (31) |
| $\left. {G(s)}_{kin} \right\rfloor_{s\to0}=\gamma=\left. G(s) \right\rfloor_{s\to0}=k_{a}+k_{b}\underset{\Rightarrow}{}\gamma=k_{a}+k_{b}$ | (32) |

**2.5 Second-order System: Parallel Subtraction**

1. Number of states$=n+1=$ 3
2. We identify the nodes and include the transitions in the diagram (see Figure 2.5). Due to the subtraction operation in the block diagram, the signal flows in the opposite direction through $G_{b}$ and the transitions between states reflect this accordingly (see Figure 2.5, center).
3. We observe that the resulting molecular kinetic scheme does not satisfy the principle of microscopic reversibility. We adjust the molecular kinetic scheme by adding a flow in the clockwise direction equal to the flow on the anticlockwise direction, and no additional independent kinetic parameters $\sigma_{i}$ are added (see Figure 2.5 right).
4. We build the system of equations

| $\left\{ \begin{matrix} \frac{dz_{2}(t)}{dt}=\sigma_{1}z_{1}\left( t \right)+\sigma_{3}z_{3}\left( t \right)-2\sigma_{2}z_{2}\left( t \right) ODE Transition 1 \\ \frac{dz_{3}(t)}{dt}=\sigma_{1}z_{1}\left( t \right)+\sigma_{2}z_{2}\left( t \right)-2\sigma_{3}z_{3}\left( t \right) ODE Transition 2 \\ \begin{matrix} y\left( t \right)=\gamma z_{2}\left( t \right) Observable Equation \\ u\left( t \right)=z_{1}\left( t \right)+z_{2}\left( t \right)+z_{3}\left( t \right) Mass Equation \end{matrix} \end{matrix} \right.$ | (33) |
| --- | --- |

5. We Laplace transform the system assuming $z_{2}\left( 0 \right)=0$ and $z_{3}\left( 0 \right)=0$ for flexibility

| $\left\{ \begin{matrix} sZ_{2}\left( s \right)=\sigma_{2}Z_{1}\left( s \right)+\sigma_{3}Z_{3}\left( s \right)-{2\sigma}_{2}Z_{2}(s) T1 \\ sZ_{3}\left( s \right)=\sigma_{1}Z_{1}\left( s \right)+\sigma_{2}Z_{2}(s)-{2\sigma}_{3}Z_{3}(s) T2 \\ \begin{matrix} Y\left( s \right)=\gamma Z_{2}\left( s \right) O \\ U\left( s \right)=Z_{1}\left( s \right)+Z_{2}\left( s \right)+Z_{3}\left( s \right) M \end{matrix} \end{matrix} \right.$ | (34) |
| --- | --- |

6. We obtain ${G(s)}_{kin}$by isolating $\frac{Y(s)}{U(s)}$ from equations T1, T2, O and M.

| ${G(s)}_{kin}=\frac{Y(s)}{U(s)}=\frac{\gamma\sigma_{1}\left( s+3\sigma_{1} \right)}{s^{2}+2s\left( \sigma_{1}+\sigma_{2}{+\sigma}_{3} \right)+3\left( \sigma_{2}\sigma_{3}+\sigma_{1}\sigma_{3}+\sigma_{1}\sigma_{2} \right)}$ | (35) |
| --- | --- |

7. From the block diagram we obtain $G(s)$

| $G(s)=\frac{\left( b_{a}+b_{b} \right){s+b}_{a}\omega_{b}{+b}_{b}\omega_{a}}{\left( s+\omega_{a} \right)(s+\omega_{b})}$ | (36) |
| --- | --- |

8. Comparing terms in ${G(s)}_{kin}$ and $G(s)$one can conclude that there is no set of kinetic parameters $\sigma_{i}$and $\gamma$such that ${G(s)}_{kin}$ and $G(s)$are equal. We therefore add an additional non-observable state (see Figure 2.5 right). By comparing terms in ${G(s)}_{kin}$ and $G(s)$ we obtain the following equations

9. We build the new system of equations

| $\left\{ \begin{matrix} \frac{dz_{2}\left( t \right)}{dt}=\sigma_{1}z_{1}\left( t \right)+\sigma_{3}z_{4}\left( t \right)-\left( \sigma_{3}+\sigma_{2} \right)z_{2}\left( t \right) ODE Transition 1 \\ \frac{dz_{3}\left( t \right)}{dt}=\sigma_{2}z_{1}\left( t \right)+\sigma_{3}z_{4}\left( t \right)-\left( \sigma_{3}+\sigma_{1} \right)z_{3}(t) ODE Transition 2 \\ \begin{matrix} \begin{matrix} \frac{dz_{4}(t)}{dt}=\sigma_{1}z_{3}\left( t \right)+\sigma_{2}z_{2}\left( t \right){-2\sigma}_{3}z_{4}\left( t \right) ODE Transition 3 \\ y\left( t \right)=\gamma z_{2}\left( t \right) Observable Equation \end{matrix} \\ u\left( t \right)=z_{1}\left( t \right)+z_{2}\left( t \right)+z_{3}\left( t \right) +z_{4}\left( t \right) Mass Equation \end{matrix} \end{matrix} \right.$ | (37) |
| --- | --- |

| 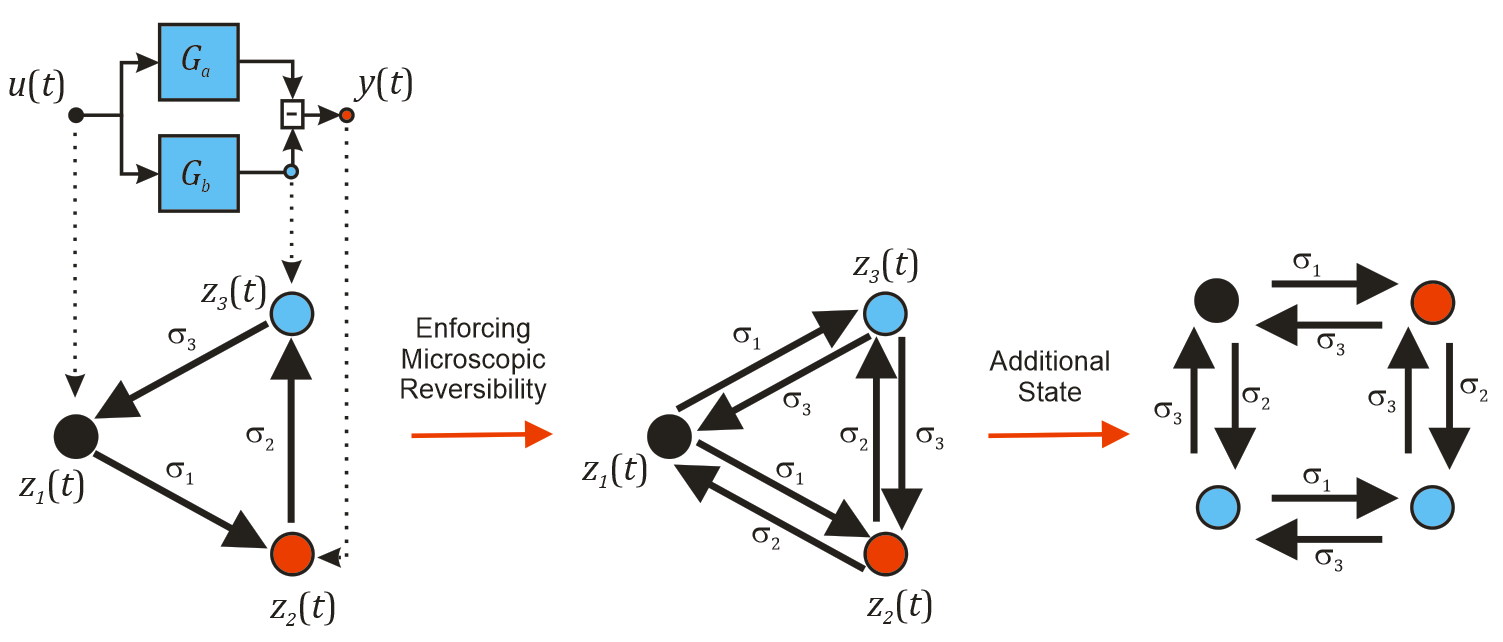 |
| --- |
| ***Figure 2.5.*** *Molecular kinetic scheme derivation of two first-order systems,* $G_{a}$ *and* $G_{b}$*, substracted in parallel. (***Left***) First molecular kinetic scheme which does not satisfy the condition of microscopic reversibility. (***Center***) Molecular kinetic scheme where microscopic reversibility has been enforced without a solution for* $\sigma_{i}$ *and* $\gamma$*. (***Right***) Molecular kinetic scheme with microscopic reversibility and an additional state with solution for the kinetic parameters* $\sigma_{i}$ *and* $\gamma$*.* |

10. We Laplace transform the system assuming $z_{2}\left( 0 \right)=0$, $z_{3}\left( 0 \right)=0$, and $z_{4}\left( 0 \right)=0$ for flexibility

| $\left\{ \begin{matrix} sZ_{2}(s)=\sigma_{1}Z_{1}\left( s \right)+\sigma_{3}Z_{4}\left( s \right)-\left( \sigma_{3}+\sigma_{2} \right)Z_{2}\left( s \right) T1 \\ sZ_{3}(s)=\sigma_{2}Z_{1}\left( s \right)+\sigma_{3}Z_{4}\left( s \right)-\left( \sigma_{3}+\sigma_{1} \right)Z_{3}(s) T2 \\ \begin{matrix} \begin{matrix} sZ_{4}(s)=\sigma_{1}Z_{3}\left( s \right)+\sigma_{2}Z_{2}\left( s \right){-2\sigma}_{3}Z_{4}\left( s \right) T3 \\ Y\left( s \right)=\gamma Z_{2}\left( s \right) O \end{matrix} \\ U(s)=Z_{1}\left( s \right)+Z_{2}\left( s \right)+Z_{3}\left( s \right)+Z_{4}\left( s \right) M \end{matrix} \end{matrix} \right.$ | (38) |
| --- | --- |

11. We obtain ${G(s)}_{kin}$by isolating $\frac{Y(s)}{U(s)}$ from equations T1, T2, O and M and simplifying the expression

| ${G(s)}_{kin}=\frac{Y(s)}{U(s)}=\frac{\gamma\left( s+\sigma_{3} \right)}{(s+\sigma_{1})(s+\sigma_{2}+\sigma_{3})}$ | (39) |
| --- | --- |

12. From the block diagram we obtain $G(s)$

| $G(s)=\frac{\left( b_{a}+b_{b} \right){s+b}_{a}\omega_{b}{+b}_{b}\omega_{a}}{\left( s+\omega_{a} \right)(s+\omega_{b})}$ | (40) |
| --- | --- |

13. We compare terms in ${G(s)}_{kin}$ and $G(s)$ obtain the following equations

| $\sigma_{1}=\omega_{a}$ | (41) |
| --- | --- |
| $\sigma_{2}=\omega_{b}-\sigma_{3}$ | (42) |
| $\sigma_{3}=\frac{b_{a}\omega_{b}+ b_{b}\omega_{a}}{b_{a}+b_{b}}$ | (43) |
| $\left. {G(s)}_{kin} \right\rfloor_{s\to0}=\frac{\gamma\sigma_{3}}{\sigma_{1}(\sigma_{2}+\sigma_{3})}=\left. G(s) \right\rfloor_{s\to0}=k_{a}+k_{b}\underset{\Rightarrow}{}\gamma=\frac{\left( k_{a}+k_{b} \right)\omega_{a}\omega_{b}}{\sigma_{3}}$ | (44) |

**3. Scalability**

To demonstrate the scalability of SYSMOLE, we present in this section its implementation for an example in which the traces arise from a third order system. Let us assume that we have a set of traces in response to a stimulus from 10 different experiments as the ones depicted in Figure 3.1. We would like to use SYSMOLE to extract information about the molecular kinetic scheme Markov-chain state network underlying these traces.

| 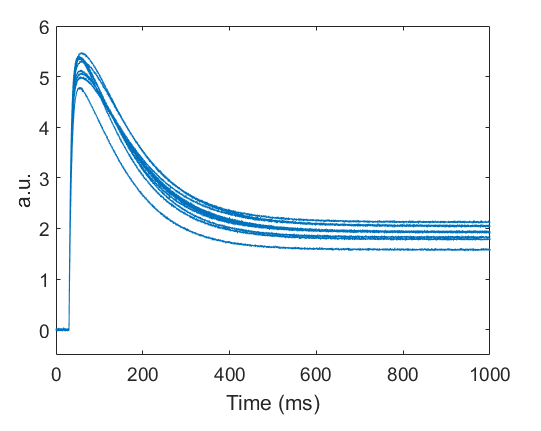 |
| --- |
| ***Figure 3.1.*** *Example of 10 simulated experimental traces arising from a third order system. Stimulus was given at time = 30 ms* |

As with real experimental traces, we do not know a priori the order of the system, which is determined by the number of poles and zeros in the transfer function obtained by the Identifier Module. It is indeed difficult to determine merely by visual inspection whether these traces are associated with a second-order or a third-order system. The implementation of the Identifier Module does not need scaling since it is already designed to detect as many processes as there may be in the trace. ARX methods have been used to successfully characterize high-order systems (up to at least order 5) [1, 2]. In practice, the real limitation to scalability arises from the sampling frequency of the data and the fastest process that can be captured at that frequency determined by the Nyquist theorem. Furthermore, the presence of noise can result in the loss of some of the poles and zeros. As mentioned in the main text, SYSMOLE is best adapted to analyze adequately-sampled traces. The next section in this document (section 4. Noise) provides an extensive noise robustness analysis of SYSMOLE and strategies to improve the error-free SNR region.

Analogous principles and methodology applied to implement the Classifier Module for second-order systems (as described in the main text) can be adapted for higher-order transfer functions. The flow chart should be expanded and the number of optimization problems increased. In our example, for all ten traces the Identifier Module extracted a third-order transfer function associated with three poles and two zeros. The task of the Classifier Module will be to discriminate among the different combinations of the configurations (cascade, feedback, and parallel) that yield third-order transfer functions with 3 poles and 2 zeros. From all combinations possible, five combinations of configurations can be described with 3 poles and 2 zeros: Cascade-Parallel (CP), Feedback-Feedback (FF), Feedback-Parallel (FP), Parallel-Feedback (PF), and Parallel-Parallel (PP) (other combinations yield three poles and one zero, or three poles and no zeros). In a similar fashion to that of second-order systems, one can implement the Classifier Module by solving five optimization problems and comparing the value of their respective cost functions ($f_{valCP}$,$f_{valFF}$, $f_{valFP}$, $f_{valPF}$, and $f_{valPP}$,) (see equations (13) and (16) in the main text), as depicted on Figure 3.2. Furthermore, it should be noted that optimization algorithms, such as the one proposed to solve the optimization problems (see *Materials and Methods*), have been successfully applied to solve high-order equations [3].

Mathematically, the transfer functions and set of equations to build the cost function in the optimization problem for each combination are as follows:

**Cascade-Parallel (CP)**: 3 poles and 2 zeros

| $G_{CP}(s)=\frac{b_{a}b_{b}\left( s+\omega_{c} \right)+b_{c}(s^{2}+2\omega_{a}\omega_{b}s+\omega_{a}\omega_{b})}{(s+\omega_{a})(s+\omega_{b})(s+\omega_{c})}$ | (45) |
| --- | --- |

This yields for CP optimization problem:

| $\left\{ \begin{matrix} B_{2}=b_{c} \\ B_{1}=b_{a}b_{b}+b_{c}(\omega_{a}+\omega_{b}) \\ B_{0}=b_{a}b_{b}\omega_{c}+b_{c}\omega_{a}\omega_{b} \\ A_{2}=\omega_{a}+\omega_{b}+\omega_{c} \\ \begin{matrix} A_{1}=\omega_{a}\omega_{b}+\omega_{c}(\omega_{a}+\omega_{b}) \\ A_{0}=\omega_{a}\omega_{b}\omega_{c} \end{matrix} \end{matrix} \right.$ | (46) |
| --- | --- |

**Feedback-Feedback (FF)**: 3 poles and 2 zeros

| $G_{FF}(s)=\frac{b_{a}\left( s+\omega_{b} \right)\left( s+\omega_{c} \right)}{(s+\omega_{a})(s+\omega_{b}+b_{b})(s+\omega_{c}{+b}_{c})}$ | (47) |
| --- | --- |

This yields for the FF optimization problem:

| $\left\{ \begin{matrix} B_{2}=b_{a} \\ B_{1}=b_{a}(\omega_{b}+\omega_{c}) \\ B_{0}=b_{a}\omega_{b}\omega_{c} \\ A_{2}=\omega_{a}+\omega_{b}+b_{b}+\omega_{c}+b_{c} \\ \begin{matrix} A_{1}=\omega_{a}{(\omega}_{b}+b_{b})+{(\omega}_{c}+b_{c})(\omega_{a}+\omega_{b}+b_{b}) \\ A_{0}=\omega_{a}{(\omega}_{b}+b_{b}{)(\omega}_{c}+b_{c}) \end{matrix} \end{matrix} \right.$ | (48) |
| --- | --- |

**Feedback-Parallel (FP)**: 3 poles and 2 zeros

| $G_{FP}(s)=\frac{b_{a}\left( s+\omega_{b} \right)\left( s+\omega_{c} \right)+b_{c}\left( s+\omega_{a} \right)\left( s+\omega_{b}+b_{b} \right)}{(s+\omega_{a})(s+\omega_{b}+b_{b})(s+\omega_{c})}$ | (49) |
| --- | --- |

This yields for the FP optimization problem:

| $\left\{ \begin{matrix} B_{2}=b_{a}+b_{c} \\ B_{1}=b_{a}\left( \omega_{b}+\omega_{c} \right)+b_{c}\left( \omega_{a}+\omega_{b}+b_{b} \right) \\ B_{0}=b_{a}\omega_{b}\omega_{c}+b_{c}\omega_{a}(\omega_{b}+b_{b}) \\ A_{2}=\omega_{a}+\omega_{b}+b_{b}+\omega_{c} \\ \begin{matrix} A_{1}=\omega_{a}{(\omega}_{b}+b_{b})+\omega_{c}(\omega_{a}+\omega_{b}+b_{b}) \\ A_{0}=\omega_{a}{(\omega}_{b}+b_{b}{)\omega}_{c} \end{matrix} \end{matrix} \right.$ | (50) |
| --- | --- |

**Parallel-Feedback (PF)**: 3 poles and 2 zeros

| $G_{PF}(s)=\frac{{(b}_{a}+b_{b})s^{2}+s[\left( {b_{a}+b_{b})\omega}_{c}+ b_{a}\omega_{b}+b_{b}\omega_{a} \right]+\omega_{c}(b_{a}\omega_{b}+b_{b}\omega_{a})}{(s+\omega_{a})(s+\omega_{b})(s+\omega_{c})}$ | (51) |
| --- | --- |

This yields for the PF optimization problem:

| $\left\{ \begin{matrix} B_{2}=b_{a}+b_{b} \\ B_{1}=b_{a}\omega_{b}+b_{b}\omega_{a}+b_{a}\omega_{c}+b_{b}\omega_{c} \\ B_{0}={(b}_{a}\omega_{b}+b_{b}\omega_{a})\omega_{c} \\ A_{2}=\omega_{a}+\omega_{b}+b_{c}+\omega_{c} \\ \begin{matrix} A_{1}=\omega_{a}\omega_{b}+(\omega_{c}{+b}_{c})(\omega_{a}+\omega_{b}) \\ A_{0}=\omega_{a}\omega_{b}(\omega_{c}+b_{c}) \end{matrix} \end{matrix} \right.$ | (52) |
| --- | --- |

**Parallel-Parallel (PP)**: 3 poles and 2 zeros

| $G_{PP}\left( s \right)=\frac{{(b}_{a}+b_{b})s+b_{a}\omega_{b}+b_{b}\omega_{a}}{\left( s+\omega_{a} \right)\left( s+\omega_{b} \right)}*\frac{b_{c}}{\left( s+\omega_{c} \right)}$ | (53) |
| --- | --- |

This yields for the PP optimization problem:

| $\left\{ \begin{matrix} B_{2}=b_{a}+b_{b}+b_{c} \\ B_{1}=b_{a}\omega_{b}+b_{b}\omega_{a}+(b_{a}+b_{b}{)\omega}_{c}+b_{c}(\omega_{a}+\omega_{b}) \\ B_{0}=\omega_{c}\left( b_{a}\omega_{b}+b_{b}\omega_{a} \right)+b_{c}\omega_{a}\omega_{b} \\ A_{2}=\omega_{a}+\omega_{b}+\omega_{c} \\ \begin{matrix} A_{1}=\omega_{a}\omega_{b}+\omega_{c}(\omega_{a}+\omega_{b}) \\ A_{0}=\omega_{a}\omega_{b}\omega_{c} \end{matrix} \end{matrix} \right.$ | (54) |
| --- | --- |

| 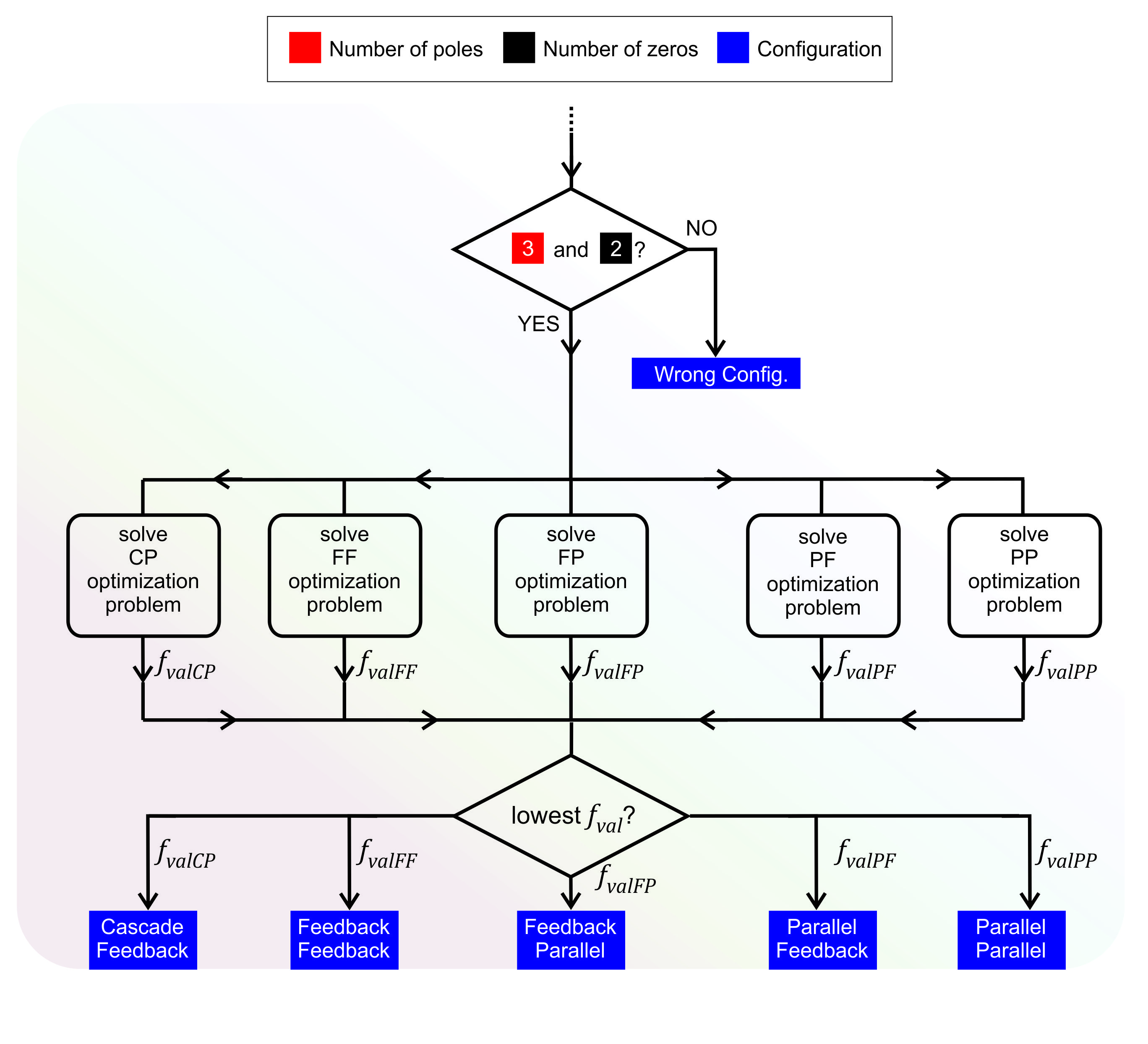 |
| --- |
| ***Figure 3.2.*** *Classifier Module flow chart implementation for third-order systems characterized by two poles and three zeros* |

Once the classifier implemented, we tested SYSMOLE’s ability to determine the right combination of configurations for a wide range of traces. To that aim, we generated synthetic traces using each of the five combinations (CP, FF, FP, PF, and PP) to which we added Gaussian noise with increasing variance. We generated 100 simulations for each noise level, with parameter values for $k_{a}$,$k_{b}$,$k_{c}$,$\tau_{a}$,$\tau_{b}$,and $\tau_{c}$ ranging as described on the table below (Table 3.1). As discussed for second-order systems (see section 1 in this document), these ranges were chosen to avoid parameter regions in which solutions for multiple combinations exist. Furthermore, visual inspection of the traces (Fig. 3.1) allows us to determine that the three processes detected by the identifier show differences in their time constants: a fast inflection first, followed by two slower ones. This information has therefore been added to the Classifier Module in the choice of these ranges.

|  | **CP** | **FF** | **FP** | **PF** | **PP** |
| --- | --- | --- | --- | --- | --- |
| $k_{a}$ | [-5,5] | [-5,5] | [-5,5] | [-5,5] | [-5,5] |
| $k_{b}$ | [-5,5] | [0,5] | [0, 5] | [-5,5] | [-5,5] |
| $k_{c}$ | [-5,5] | [0,5] | [-5,5] | [0,5] | [-5,5] |
| $\tau_{a}$ | [2,6] | [2,6] | [2,6] | [2,6] | [2,6] |
| $\tau_{b}$ | [30,100] | [30,100] | [30,100] | [30,100] | [30,100] |
| $\tau_{c}$ | [300,500] | [300,500] | [300,500] | [300,500] | [300,500] |

***Table 3.1***

As one could expect, each combination yields signals which vary greatly in terms of their power. To compare the robustness to noise among configurations, we normalized the signal-to-noise ratio (SNR) of each signal by dividing it by the SNR for that combination to a low level of Gaussian noise with standard deviation of 10^-5^. The results indicate a probability of error that increases sharply at a normalized SNR between 0.5 and 0.6 for all the combinations (the traces in Figure 3.1 have an equivalent normalized SNR of approximately 0.8). Applying SYSMOLE to the traces in our example (Fig. 3.1) yielded the combination PF for all ten traces.

| 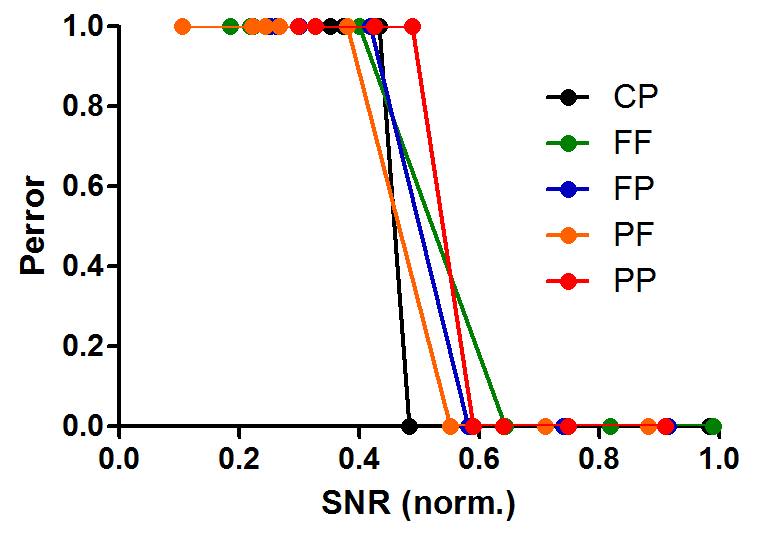 |
| --- |
| ***Figure 3.3.*** *Probability of error as a function of normalized SNR (see text) in the Classifier Module for combinations with three poles and two zeros. (CP) Cascade-Parallel, (FF) Feedback-Feedback, (FP) Feedback-Parallel, (PF) Parallel-Feedback, and (PP) Parallel-Parallel.* |

Finally, the analytical methodology underlying the molecular kinetic converter (MKC) described for second-order systems can be extended to higher order systems with more than two processes. The three canonical second-order configurations (i.e. cascade, feedback, and parallel) can be used as building units to derive the molecular kinetic schemes associated with more complex block diagrams and transfer functions. From the solutions for $k_{a}$,$k_{b}$,$k_{c}$,$\tau_{a}$,$\tau_{b}$,and $\tau_{c}$ (Table 3.2) we observe that both $k_{a}$, and $k_{b}$ are positive, which indicates that the parallel configuration will be in the addition configuration. For the PF combinations determined by the Classifier Module we would find the following molecular kinetic scheme (Figure 3.4). The values for the $\sigma_{i}$ can be obtained following the steps described in the previous section and solving the corresponding system of equations either analytically, or given the increased complexity of the system of equations, using optimization techniques.

| 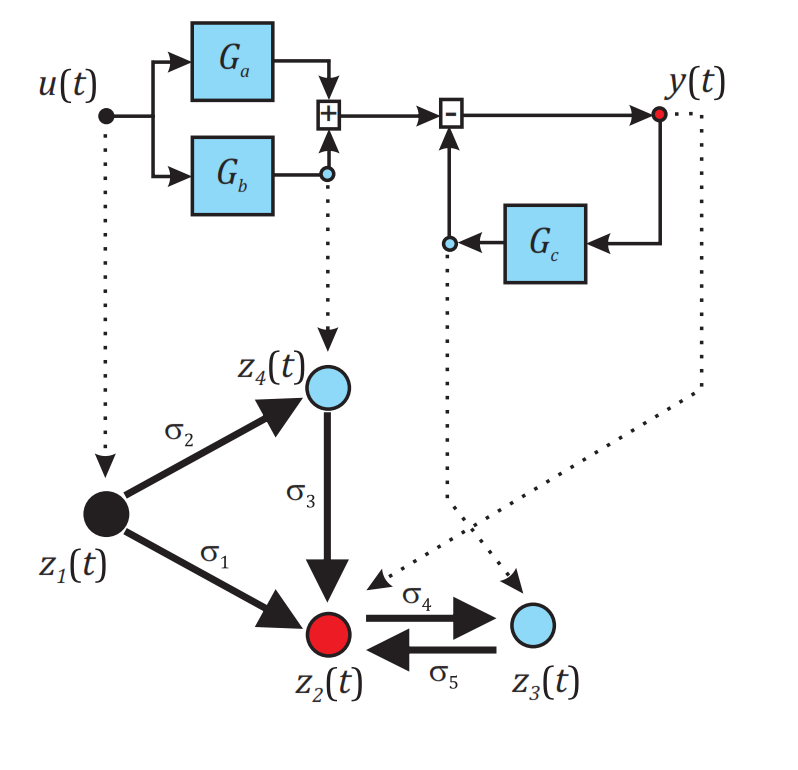 |
| --- |
| ***Figure 3.4.*** *Molecular Kinetic Scheme associated with the Parallel-feedback (PF) third-order combination with the Parallel configuration in addition.* |

The systems of differential equations that describes the PF molecular kinetic scheme is:

| $\left\{ \begin{matrix} \frac{dz_{4}\left( t \right)}{dt}=\sigma_{2}z_{1}\left( t \right)-\sigma_{3}z_{4}\left( t \right) ODE Transition 1 \\ \frac{dz_{2}\left( t \right)}{dt}=\sigma_{3}z_{4}\left( t \right)+\sigma_{1}z_{1}\left( t \right)+\sigma_{5}z_{3}\left( t \right)-\sigma_{4}z_{2}\left( t \right) ODE Transition 2 \\ \begin{matrix} \begin{matrix} \frac{dz_{3}(t)}{dt}=\sigma_{4}z_{2}\left( t \right)-\sigma_{5}z_{3}\left( t \right) ODE Transition 3 \\ y\left( t \right)=\gamma z_{2}\left( t \right) Observable Equation \end{matrix} \\ u\left( t \right)=z_{1}\left( t \right)+z_{2}\left( t \right)+z_{3}\left( t \right) +z_{4}\left( t \right) Mass Equation \end{matrix} \end{matrix} \right.$ | (55) |
| --- | --- |

We Laplace transform the system assuming $z_{2}\left( 0 \right)=0$, $z_{3}\left( 0 \right)=0$, and $z_{4}\left( 0 \right)=0$ for flexibility

| $\left\{ \begin{matrix} sZ_{4}\left( s \right)=\sigma_{2}Z_{1}\left( s \right)-\sigma_{3}Z_{4}\left( s \right) T1 \\ sZ_{2}\left( s \right)=\sigma_{3}Z_{4}\left( s \right)+\sigma_{1}Z_{1}\left( s \right)+\sigma_{5}Z_{3}\left( s \right)-\sigma_{4}Z_{2}\left( s \right) T2 \\ \begin{matrix} \begin{matrix} sZ_{3}\left( s \right)=\sigma_{4}Z_{2}\left( s \right)-\sigma_{5}Z_{3}\left( s \right) T3 \\ Y\left( s \right)=\gamma Z_{2}\left( s \right) O \end{matrix} \\ U\left( s \right)=Z_{1}\left( s \right)+Z_{2}\left( s \right)+Z_{3}\left( s \right)+Z_{4}\left( s \right) M \end{matrix} \end{matrix} \right.$ | (56) |
| --- | --- |
|  |  |

We obtain ${G(s)}_{kin}$by isolating $\frac{Y(s)}{U(s)}$ from equations T1, T2, O and M and simplifying the expression

| ${G(s)}_{kin}=\frac{\gamma[\sigma_{1}s^{2}+s\left[ \sigma_{3}\left( \sigma_{2}+\sigma_{1} \right)+\sigma_{5}\sigma_{1} \right]+\sigma_{5}\sigma_{3}\left( \sigma_{2}+\sigma_{1} \right)]}{s^{3}+s^{2}\left( \sigma_{5}+\sigma_{4}+\sigma_{3}+\sigma_{1}+\sigma_{4}\sigma_{2} \right)+s\left[ \sigma_{3}\left( \sigma_{1}+\sigma_{2}+\sigma_{5}+\sigma_{4} \right)+\left( \sigma_{5}+\sigma_{4} \right)\left( \sigma_{1}+\sigma_{2} \right) \right]+(\sigma_{2}+\sigma_{1})\sigma_{3}(\sigma_{5}+\sigma_{4})}$ | (57) |
| --- | --- |

We solve for $\sigma_{1}$,$\sigma_{2}$.$\sigma_{3}$,$\sigma_{4}$, $\sigma_{5}$, and $\gamma$ computational by comparing it to the coefficients $B_{2}$,$B_{1}$.$B_{0}$,$A_{2}$, $A_{1}$, and $A_{0}$ from the transfer function obtained by the Identifier Module.


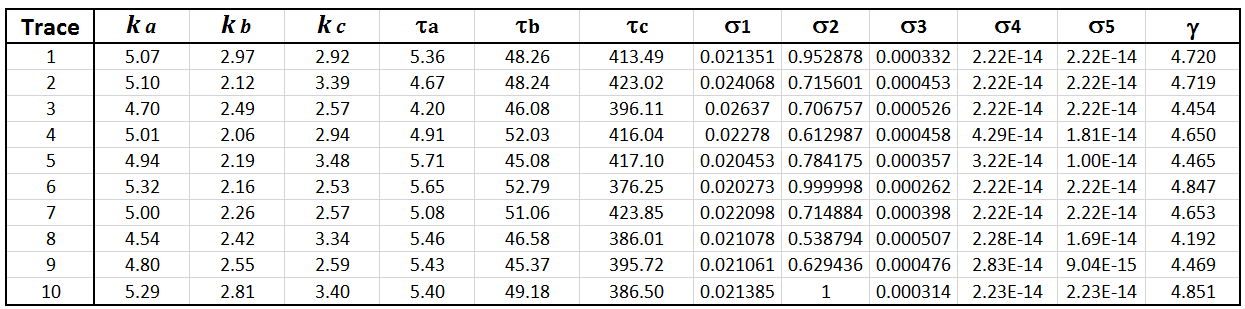


***Table 3.2***

**4. Noise**

**4.1 Brownian noise**

In the main text we explored the effect of added Gaussian noise to the trace on the probability of error of detecting the right configuration from the trace (Fig.5). We decided to test the robustness of SYSMOLE to added Brownian noise, which is common in the diffusion of molecules in anisotropic environments, such as cellular membranes [4,5]. We used the Synthetic Trace Simulator to generate similar traces to those of the L-type calcium and heteromeric GPCR experiments, add noise, and test the ability of SYSMOLE to uncover the correct configuration. Specifically, we added Brownian noise with amplitudes ranging from 0.001 to 1.5 to traces generated by two processes with parameters $k_{a}$ = - 5, $k_{b}$ = 3, $\tau_{a}$ = 5 ms, and $\tau_{b}$ = 100 ms either in feedback or in parallel (Figure 4.1).

| 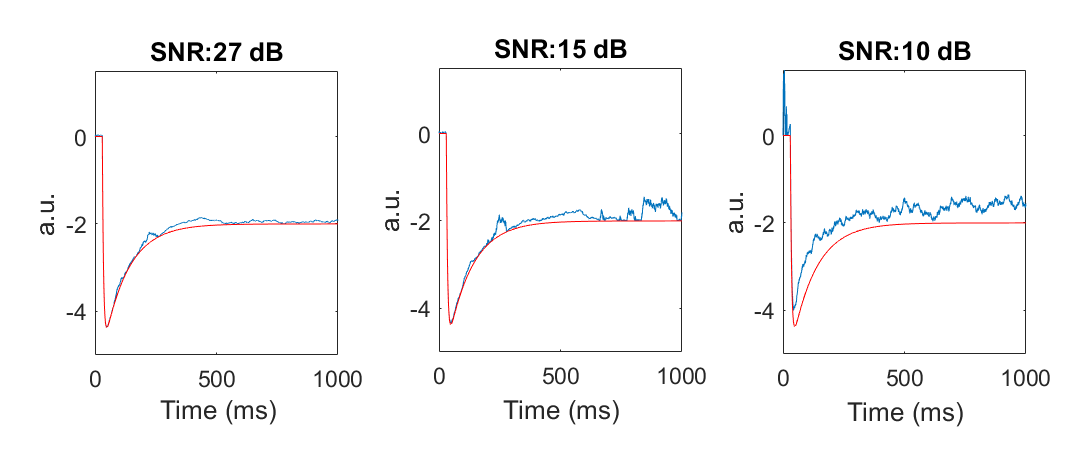 |
| --- |
| ***Figure 4.1.*** *Example of traces with added Brownian noise of amplitude 0.25, 0.75, and 1.25 respectively. Traces are the result of a parallel subtraction configuration with* $k_{a}$ *= - 5,* $k_{b}$ *= 3,* $\tau_{a}$*= 5 ms, and* $\tau_{b}$*=100 ms. Red depicts the trace without noise.* |

We ran 100 simulations for each level of Brownian noise added, and computed the probability of error as the number of simulations correctly assigned divided by the total number of simulations. The results indicate that SYSMOLE is robust to the presence of Brownian noise in these traces, with probabilities of error starting to increase at a SNR of 18 dB for the parallel configuration and 14 dB for the feedback configuration.

| 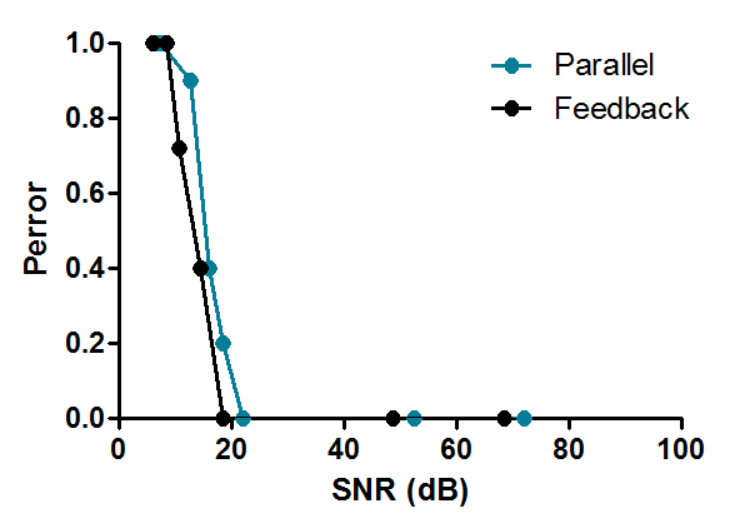 |
| --- |
| ***Figure 4.2.*** *Probability of error in assigning the right configuration by SYSMOLE as a function of SNR. Each data point represents 100 simulations with parameters* $k_{a}$ *= - 5,* $k_{b}$ *= 3,* $\tau_{a}$ *= 5 ms and* $\tau_{b}$ *= 100 ms. Classifier boundary conditions used are* $\tau_{a}\in$ *[1, 10] ms,* $\tau_{b}\in$ *[50, 250] ms,* $k_{a}\in$ *[−10, 10], and* $k_{b}\in$ *[−10, 10] for the parallel problem and* $k_{b}\in$ *[0, 10] for the feedback problem since combinations in feedback with* $k_{b}$ *< 0 are unstable* |

**4.2 Improving the SNR requirement for error-free classification.**

The results depicted on the main text indicate that, in the presence of additive Gaussian noise, the probability of error in determining the right configuration sharply increased for signal-to-noise ratios below 25 dB and 22 dB for the parallel and feedback configurations, respectively. One possible strategy to reduce the probability of error would be to filter the trace prior to the application of SYSMOLE. Filtering increases the SNR and allows the Identifier Module to successfully detect the poles and zeros, and the Classifier Module to accurately determine the configuration.

To illustrate the use of this strategy, we applied a moving-average filter to the traces generated by the Synthetic Trace Simulator in the main text to study the robustness of SYSMOLE to Gaussian noise in the second-order parallel subtraction and feedback configurations. Filtering the traces resulted in an overall increase in SNR of 6.7 dB and 6.5 dB for the parallel and feedback configurations, respectively (Figures 4.3 and 4.4).

| 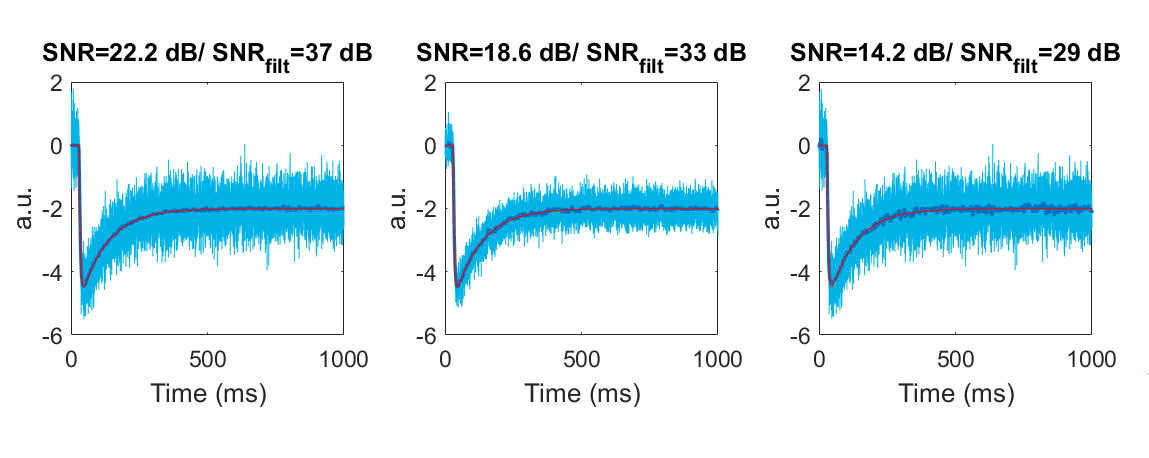 |
| --- |
| ***Figure 4.3.*** *Illustration of the improvement in signal-to-noise ratio achieved by filtering. Traces are the result of two processes combined through a parallel subtraction configuration with* $k_{a}$ *= - 5,* $k_{b}$ *= 3,* $\tau_{a}$*= 5 ms, and* $\tau_{b}$*=100 ms. Red depicts the trace without noise, cyan the traces with noise, and dark blue the traces filtered traces with a moving-average filter with a window of 3 ms.* |

| 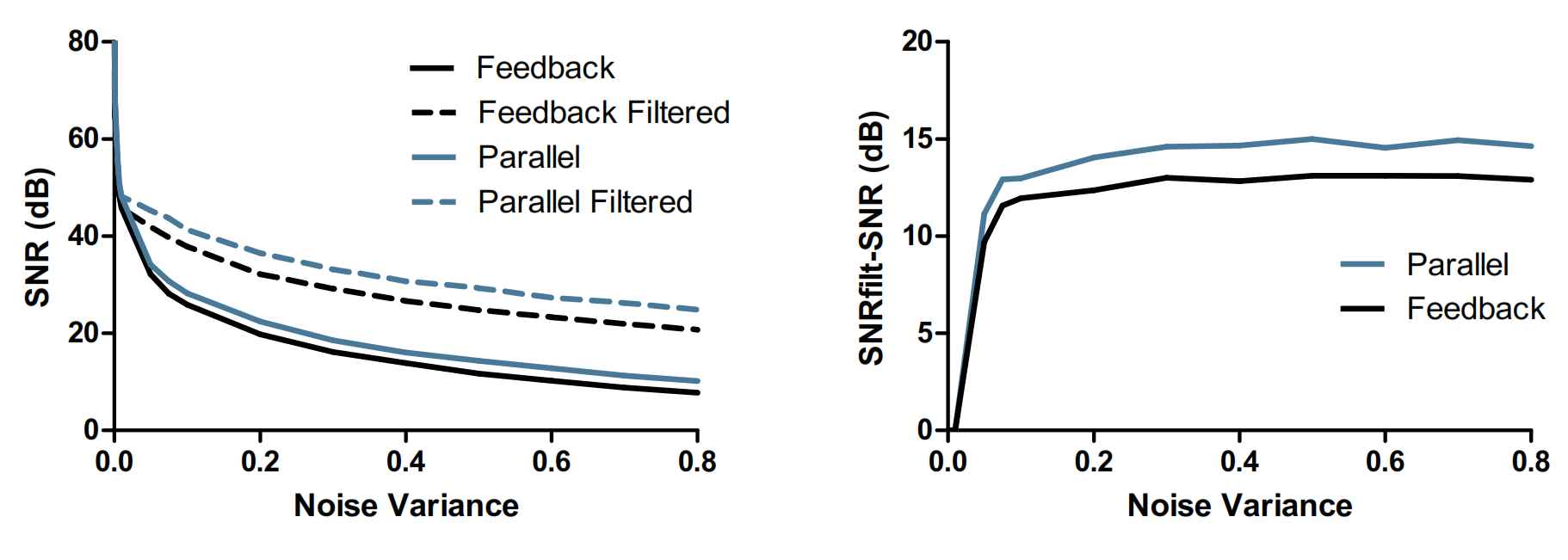 |
| --- |
| ***Figure 4.4.*** *Improvement in signal-to-noise ratio (SNR) by filtering the trace with a moving-average filter of window 3 ms prior to application of SYSMOLE in second-order parallel and feedback configurations.* |

In addition, this improvement in SNR translated into a decrease in the minimum SNR required to guarantee error-free classification (Figure 4.5). A significant improvement when filtering prior to application to SYSMOLE is also observed for third-order systems with three poles and two zeros (Figure. 4.6).

| 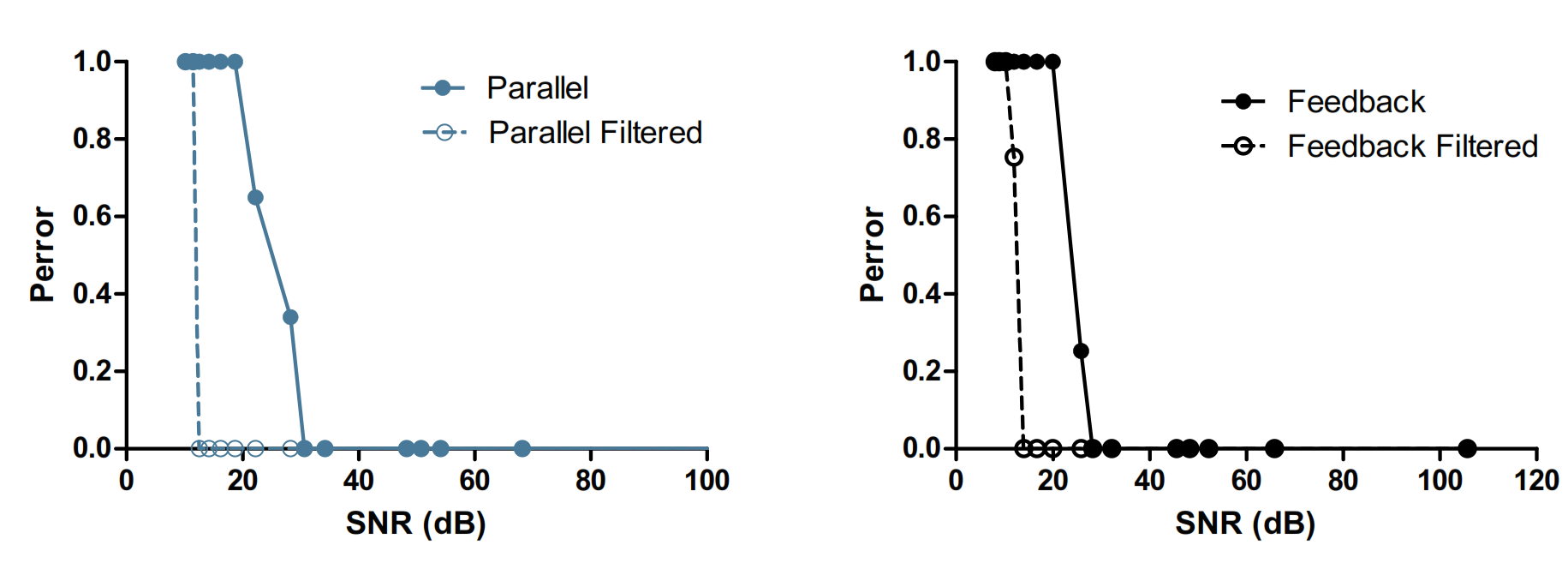 |
| --- |
| ***Figure 4.5.*** *Improvement in probability of error (Perror) in assigning the correct configuration by filtering the trace with a moving-average filter of window 3 ms prior to application of SYSMOLE for the second-order parallel and feedback configurations.* |

| 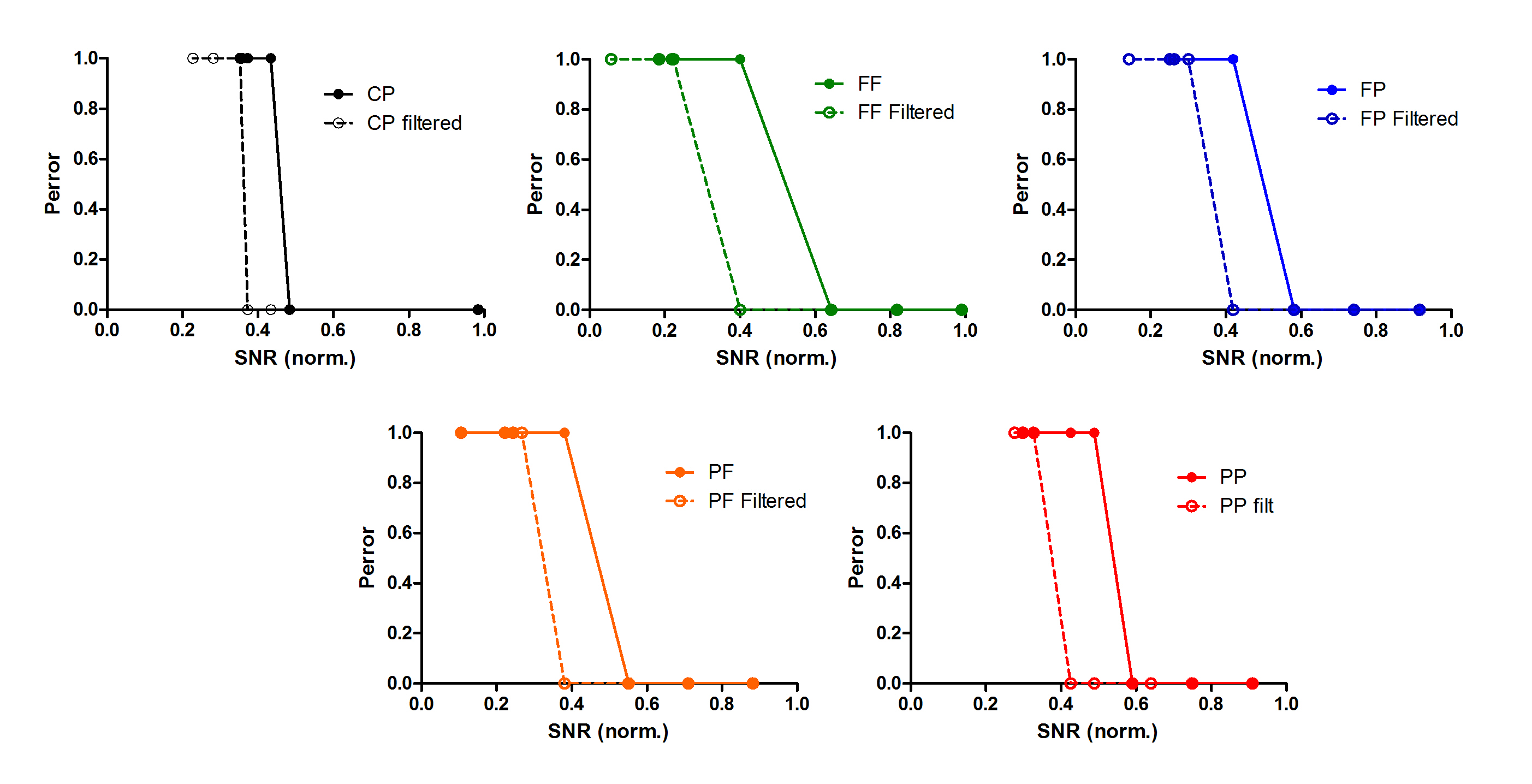 |
| --- |
| ***Figure 4.6.*** *Improvement in probability of error (Perror) in assigning the correct configuration by filtering the trace with a moving-average filter of window 3 ms prior to application of SYSMOLE in third-order systems with three poles and two zeros.* |

The SYSMOLE Matlab toolbox that accompanies this work can be found in Matlab Central (<http://www.mathworks.com/matlabcentral>/fileexchange/61465-sysmole) The toolbox allows the user to simulate his or her own experimental traces and determine the levels of noise for which SYSMOLE performs at low probability of error for the biological system under study. Multiple pre-processing strategies exist to eliminate noise in the traces [6], and the most adequate for each type of experimental trace should be determined if the SNR of the experimental traces are below a value that will provide error-free detection.

**4.3 Application of SYSMOLE to uncover molecular kinetic schemes in the presence of single-cell gene expression noise.**

In order to explore the versatility of SYSMOLE to tackle non-classical types of noise, we decided to test whether we could use SYSMOLE to tease out different gene regulatory mechanisms in the presence of cell-to-cell gene induction noise. Gene expression in response to a given stimulus varies among cells, even when the cell population is homogeneous [7-9]. Our previous studies have successfully utilized single-cell single-molecule techniques to characterize this cell-to-cell variability or noise in the induction of the interferon beta gene (*Ifnb1*), a key cytokine involved in innate immune responses [10]. Specifically, we established that cell-to-cell variability in the rate of induction (in mRNA molecules per hour) of *Ifnb1* in dendritic cells exposed to a lipopolysaccharide (LPS) present in bacterial walls can be characterized by a gamma distribution with size and shape parameters values of 3 and 2.5 respectively (58).

In order to translate this finding to our framework, we described gene induction as a first-order process $G_{a}(s)$ characterized by a time constant $\tau_{a}$ = 3 hours, and a $k_{a}$ that varies for each cell as follows (Figure 4.8):

| $b_{a}\in\gamma\left( 3,2.5 \right)$ | (58) |
| --- | --- |
| $k_{a}=b_{a}\tau_{a}$ | (59) |

| 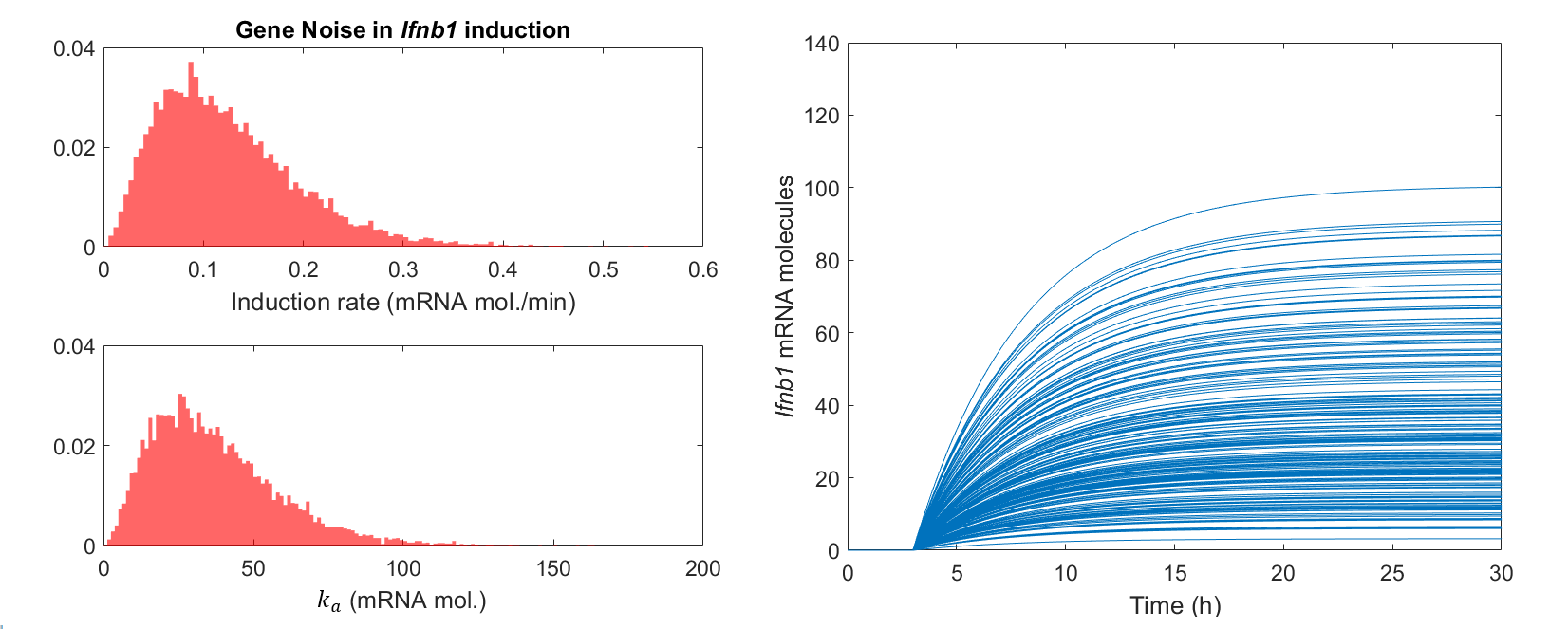 |
| --- |
| ***Figure 4.8.*** *Cell-to-cell variability in Ifnb1 gene induction. (derived from experimental measurements [10]). Distribution of induction rate (*$b_{a})$ *and gain parameter (*$k_{a})$ *and representative traces for Ifnb1 induction.* |

We included a second first-order process responsible for regulating gene expression in a feedback or parallel subtraction configuration $G_{b}(s)$, with parameters $k_{b}$ = -25 mRNA mol. for the parallel configuration and 25 mRNA mol. for the feedback configuration and $\tau_{b}$= 16.67 hours (1000 min). Molecularly, one could potentially interpret the gene regulatory mechanisms in these schemes in terms of the inhibition of *Ifnb1* expression by a co-expressed gene, or the inhibition of *Ifnb1* by other pathogen factors [11]. We then measured the ability of SYSMOLE to recognize the underlying gene regulatory mechanism in the presence of cell-to-cell variability by testing in how many cells, out of 1000, SYSMOLE would obtain the correct configuration. SYSMOLE showed to be robust to cell-to-cell variability of *Ifnb1* induction expression with error probabilities of 0 and 0. 009 for the feedback and the parallel subtraction configurations, respectively (Figure 4.9)

Similar limitations to those described previously apply for cell-to-cell variability noise. First, sampling frequency should be available to capture the fastest process. Secondly, the gene regulation process should be working at a slower rate than gene induction. Together these results suggest that SYSMOLE is also robust to cell-to-cell variability noise.

| 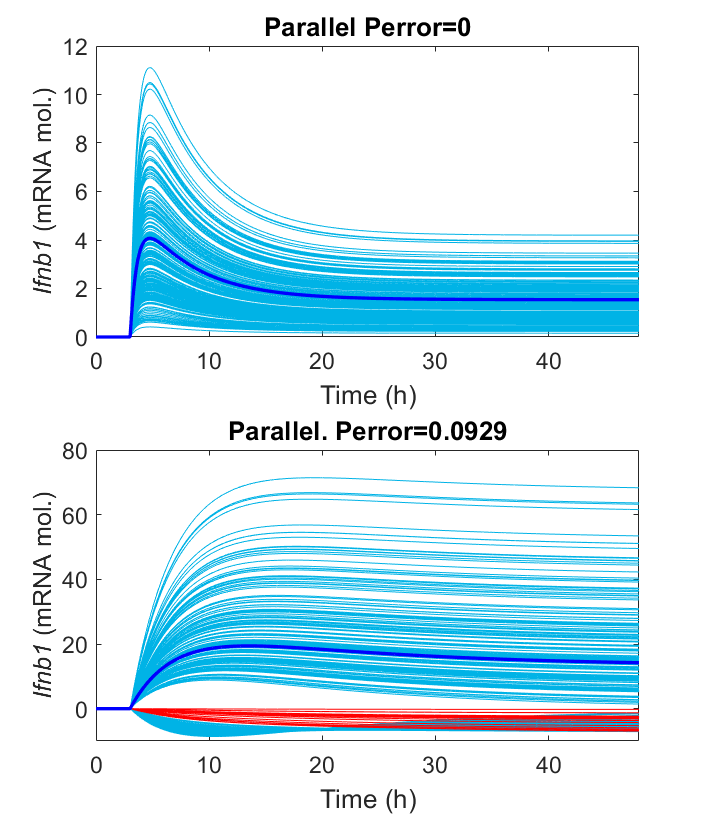 |
| --- |
| ***Figure 4.9.*** *Traces and probability of error associated with a potential gene-regulatory mechanism described by a feedback or a parallel subtraction scheme in which cell-to-cell variability noise is included following the noise model described in Figure. 4.8. Parameters are* $k_{b}$*=25 for feedback and* $k_{b}$*=-25 for parallel.* $\tau_{b}$*=100 min. Classifier parameters are (*$\tau_{a}\in$ *[1,50],* $\tau_{b}\in$ *[75,250],* $k_{a}\in$ *[-10,10],* $k_{b}\in$*[-100,100] for parallel subtraction and* $k_{b}\in$ *[0,100] for feedback.* |

**References**

1. Ljung L. (1999) System identification. theory for the user. 2nd ed.: Prentice Hall.

2. Söderström T, Fan H, Carlsson B, Bigi S. (1997) Least squares parameter estimation of continuous-time ARX models from discrete-time data. IEEE Transaction on Automatic Control 42, NO.5.

3. Dennis J, Vicente L. (1996) Trust-region interior-point algorithms for minimization problems with simple bounds. Applied Mathematics and Parallel Computing.

4.    Astumian RD. (1997) Thermodynamics and kinetics of a brownian motor. Science 276: 917-922.

5.    Astumian RD, Derenyi I. (1998) Fluctuation driven transport and models of molecular motors and pumps. Eur Biophys J 27: 474-489.

6.    Anderson BDO, Moore JB. (2005) Optimal filtering. New York: Dover. 349 p.

7.    Elowitz MB, Levine AJ, Siggia ED, Swain PS. (2002) Stochastic gene expression in a single cell. Science 297: 1183-1186. 10.1126/science.1070919 [doi].

8.    Blake WJ, KAErn M, Cantor CR, Collins JJ. (2003) Noise in eukaryotic gene expression. Nature 422: 633-637. 10.1038/nature01546 [doi].

9.     Maheshri N, O'Shea EK. (2007) Living with noisy genes: How cells function reliably with inherent variability in gene expression. Annu Rev Biophys Biomol Struct 36: 413-434. 10.1146/annurev.biophys.36.040306.132705 [doi].

10.   Patil S, Fribourg M, Ge Y, Batish M, Tyagi S, et al. (2015) Single-cell analysis shows that paracrine signaling by first responder cells shapes the interferon-beta response to viral infection. Sci Signal 8: ra16. 10.1126/scisignal.2005728 [doi].

11.   Fribourg M, Hartmann B, Schmolke M, Marjanovic N, Albrecht RA, et al. (2014) Model of influenza A virus infection: Dynamics of viral antagonism and innate immune response. J Theor Biol 351: 47-57. 10.1016/j.jtbi.2014.02.029 [doi].
